# Red-Emitting Dithienothiophene *S*,*S*-Dioxide Dyes for Cellular Membrane Staining

**DOI:** 10.3390/ma16051806

**Published:** 2023-02-22

**Authors:** Aneta Rzewnicka, Jerzy Krysiak, Róża Pawłowska, Remigiusz Żurawiński

**Affiliations:** 1 Division of Organic Chemistry, Centre of Molecular and Macromolecular Studies, Polish Academy of Sciences, Sienkiewicza 112, 90-363 Lodz, Poland; 2 Division of Bioorganic Chemistry, Centre of Molecular and Macromolecular Studies, Polish Academy of Sciences, Sienkiewicza 112, 90-363 Lodz, Poland

**Keywords:** fluorescence, fluorescent dyes, membrane staining, dithienothiophene

## Abstract

A series of dithienothiophene *S*,*S*-dioxide (**DTTDO**) dyes was designed, synthesized, and investigated for their suitability in fluorescent cell imaging. Synthetized (D-π-A-π-D)-type **DTTDO** derivatives have molecule lengths close to the thickness of the phospholipid membrane, and they contain on both ends two positively charged or neutral polar groups to increase their solubility in water and to ensure simultaneous interaction with polar groups of the inner and outer part of the cellular membrane. **DTTDO** derivatives exhibit absorbance and emission maxima in the 517–538 nm and 622–694 nm range, respectively, and a large Stokes shift up to 174 nm. Fluorescence microscopy experiments revealed that these compounds selectively intercalate into cell membranes. Moreover, a cytotoxicity assay conducted on a model human live cells indicates low toxicity of these compounds at the concentrations required for effective staining. With suitable optical properties, low cytotoxicity, and high selectivity against cellular structures, **DTTDO** derivatives are proven to be attractive dyes for fluorescence-based bioimaging.

## 1. Introduction

The plasma membrane, also called the cell membrane, is an indispensable element of all living cells and constitutes a natural semipermeable barrier between intracellular components and the outside environment. The cell membrane mainly consists of a lipid bilayer (made up of two layers of phospholipids), cholesterols, proteins, glycoproteins, and glycolipids. The proportions between each component are not constant but depend on the type of cell, its stage of development, and changes in the environment [1]. They are also responsible for different cell membrane functions. While cholesterol incorporated in the lipid bilayer is responsible for maintaining appropriate membrane fluidity at different conditions, proteins, glycoproteins, and glycolipids play an important role in cellular membrane functions essential for cell survival and its communication with the surrounding [2]. Thus, besides a structural protection and separation function, the plasma membrane also regulates the transport of substances in and out of the cell, controls metabolic processes, is involved in cell signaling and apoptotic and nonapoptotic cell death processes, cell-cell communication, as well as maintaining intracellular homeostasis [3,4,5,6,7]. The aberrations in plasma membrane functions and properties can be an essential indicator of the cell condition and numerous diseases [2], hence visualization and monitoring of these changes are of great importance from the viewpoint of an early medical diagnosis and basic research in cell biology.

**Figure 1:**
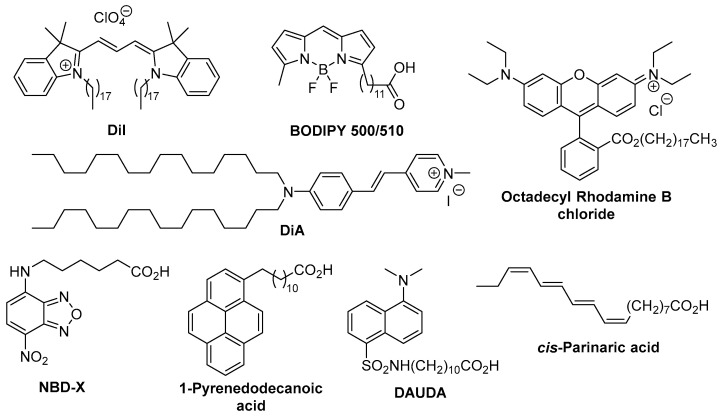
Representative commercially available cell membrane probes.

Among the methods used for lipid membrane analysis, fluorescent imaging turned out to be one of the most effective techniques, because of its high sensitivity, non-destructive nature, and capability to monitor cellular processes in real-time [8]. From the large toolbox of fluorescent probes encompassing fluorescent proteins [9], quantum dots (QDs) [10,11], and small organic dyes [12,13], especially the last one, gained the greatest popularity in membrane staining (Figure 1). This can be mainly attributed to their low cost of production, good chemical stability, easy tuning of optical and physical properties, and ability to be precisely located in specific parts of cell membranes. Moreover, due to their smaller size than fluorescent proteins and QDs, small organic dyes disturb much less membrane-dependent cellular processes.

Although the strategy in the construction of membrane-specific fluorescent probes is rather simple and based on the introduction of a hydrophilic head and hydrophobic tail to the fluorophore, their practical realization is not trivial. For example, compounds with a high lipophilic character can easily cross the plasma membrane and gather in lipid drops [14] or other internal membranes. Additionally, low solubility in the biological environment may result in their precipitation before reaching the membrane. Despite good water solubility and membrane affinity fluorescent probes should also exhibit high quantum efficiency under physiological conditions, high photostability, and broadly tuneable absorption and emission properties. Dyes active in the red or near-infrared (NIR) region are particularly interesting and highly demanded [15,16]. Advantages include significantly reduced autofluorescence from the biological sample and enhanced tissue penetration depth. Moreover, the long excitation wavelength decreases the photodamage of biological material. Another aspect that should be addressed at the design stage of the fluorescent markers is their potential fluorogenic character [17,18]. Fluorescent markers which do not exhibit fluorescence in water but are highly fluorescent in lipid membranes simplify the staining procedures and ensure observation without interference from the non-bounded probe. The presence of additional functional groups in the fluorescent probes which enables their further bioconjugation is also an advantage. Until the present day, a lot of membrane-specific fluorophores have been synthesized [19,20,21,22,23,24,25,26,27,28,29,30,31,32,33,34,35,36,37,38,39,40,41,42] based on such fluorescent scaffolds as benzothiadiazole [20,21], coumarin [21,22], BODIPY [23,24,25], squaraine [26], Nile Red [27], chromone [28], perylene [29], fluorene [30], styrylpyridinium salts [31,32,33,34], nitrobenzodiazole [35], oligo and polythiophenes [36,37,38], cyanine [39], oxazolopyridine [40] or aminostyryl derivatives [41,42]. Unfortunately, many of the commercially available fluorescent dyes have some disadvantages which reduce their application potential. For example, the problems with low solubility and the tendency to undergo aggregation are difficulties that are often encountered in the case of long-chain cyanines (such as Dil, DiR, and DiO) [43] and squaraine dyes [44]. Additionally, squaraine dyes are also highly chemically unstable in a biological environment. On the other hand, despite strong fluorescence in the far red/NIR region and good photostability, cyanine dyes exhibit intrinsically small Stokes shift that limits their application in multicolor bioimaging and may produce excitation and scattered light interferences. Therefore, new, non-toxic, photostable, and biocompatible fluorogenic markers with a high photoluminescence quantum yield and large Stokes shift are highly desirable.

**Figure 2:**
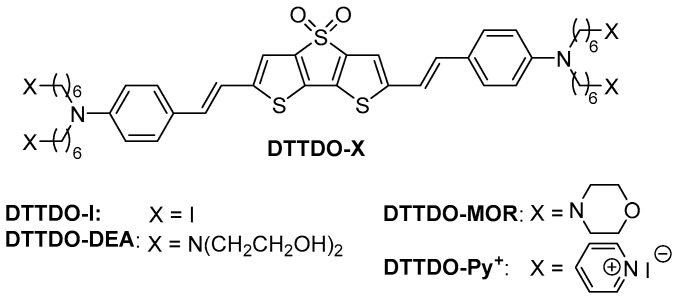
Structures of synthesized **DTTDO** derivatives.

In the present manuscript, we report a concise and efficient synthesis of a new class of dithieno [3,2-*b*:2′,3′-*d*]thiophene 4,4-dioxide (**DTTDO**) derivatives as highly selective fluorogenic red emitting plasma membrane probes (Figure 2). These compounds have a D-π-A-π-D architecture, where electron-donating dialkylaminophenyl groups (D) are connected with a strong electron-accepting **DTTDO** moiety (A) via a conjugated π linker. To ensure water solubility and simultaneous electrostatic interaction with a polar inner and outer part of the plasma membrane, **DTTDO** fluorophores are equipped with hydrophilic groups X at both ends. The distance between these terminal groups is about 3.6 nm and it roughly corresponds to the thickness of the phospholipid membrane. Investigation of the optical properties, cytotoxicity determined against selected human cell types, and the application of **DTTDO** fluorophores to plasma membrane staining in living cells, are also presented.

## 2. Results and Discussion

### 2.1. Synthesis of DTTDO Dyes

**Scheme 1:**
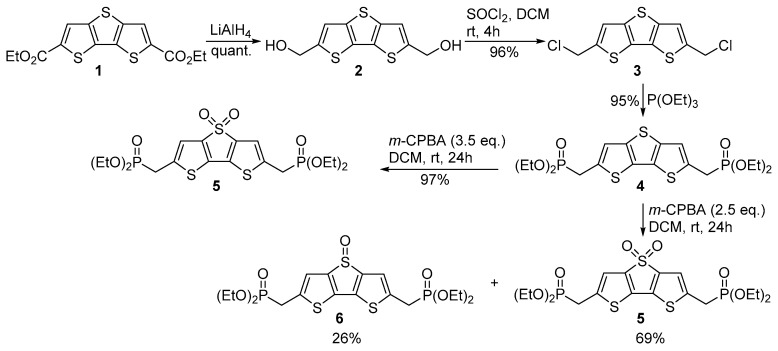
Synthesis of bisphosphonate **5**.

The synthesis of **DTTDO** derivatives started with commercially available diester **1** (also simply accessible from thiophene via a known three-step procedure [45]) (Scheme 1). The reduction of diester **1** with LiAlH_4_ followed by chlorination of crude diol **2** formed with an excess of thionyl chloride gave dichloride **3** in 96% overall yield. Heating of compound **3** with an excess of P(OEt)_3_ resulted in the formation of bisphosphonate **4**, which in the next step was subjected to the oxidation reaction with 3.5 eq of *m*-CPBA at room temperature. The oxidation process proceeded in a highly regioselective manner to afford as a sole product sulfone **5** in 97% yield. When the same reaction was conducted with a smaller excess of *m*-CPBA (2.5 eq), next to sulfone **5** sulfoxide **6** was isolated in 26% yield.

Oxidation of the sulfur atom in the central thiophene ring of bisphosphonate **4** strongly affects its optical and electronic properties (Table 1). As evidenced by fluorescence and UV-VIS measurements, the maximum absorption (λ_abs_) and emission (λ_em_) wavelengths of sulfoxide **6** are red-shifted by 60 and 130 nm, respectively, in comparison to the unoxidized counterpart. On the other hand, the oxidation of sulfoxide **6** to sulfone **5** has a much lower impact on the change of its optical properties and results in a small blue shift of λ_abs_ (8 nm) and λ_em_ (27 nm). Absorption and emission spectra of bisphosphonates **4**–**6** are available in Supplementary Materials.

**Figure 3:**
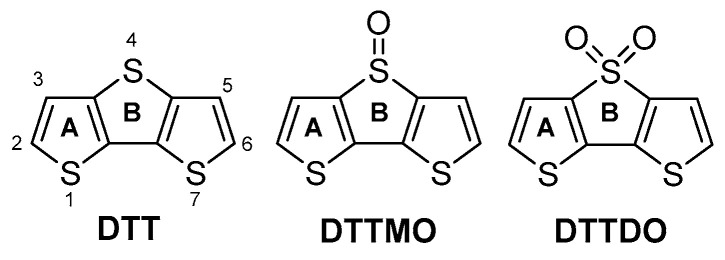
Structure, ring letters (A, B), and atom numbering of **DTT** and its oxidized derivatives.

The origin of the regioselectivity in the oxidation of **4** can be elucidated by analysis of local aromaticity and local reactivity descriptors. For this purpose, DFT calculations of dual descriptors (∆f(r)) [46], nucleus-independent chemical shifts (NICS) [47], and the geometry-based harmonic oscillator model of aromaticity (HOMA) [48], for model compounds **DTT**, **DTTMO**, and **DTTDO** were conducted (Figure 3). As indicated by local aromaticity indices the middle thiophene ring in **DTT** is less aromatic than ring A and it could be expected that this ring should undergo oxidation (Table 2). A similar conclusion can be drawn from the analysis of dual descriptors which shows that the S(4) sulfur atom has the highest negative value (−0.133) which makes it the most susceptible place for an electrophilic attack in a whole **DTT** moiety. Sulfur oxidation induces a change in the geometry of the planar thiophene ring. In **DTTMO** the oxidized thiophene ring has an envelope conformation with the sulfur and oxygen atoms directed toward the opposite sides of the plane defined by the carbon skeleton. Lower values of local aromatic indices for ring B in **DTTMO** than for the corresponding ring in **DTT** indicate that the observed deviation from planarity by about 8 deg is sufficiently high to hamper effective electron delocalization in this ring and in consequence, to reduce its aromatic character. Further oxidation proceeds on the same (less stabilized) thiophene ring and leads to the planar thiophene dioxide which has an antiaromatic character. Sulfur oxidation also exerts influence on the molecular frontier orbitals. Their energy decreases with chemical oxidation. Because the decrease of the LUMO orbital energy is higher than that of the HOMO orbital energy, it results in a smaller energy gap compared to the non-oxidized counterpart.

**Scheme 2:**
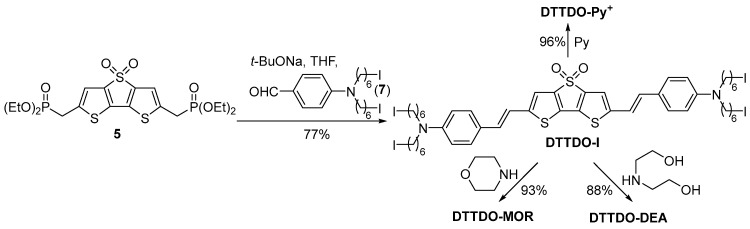
Synthesis of **DTTDO** derivatives.

The reaction sequence leading from bisphosphonate **5** to **DTTDO**-based dyes is presented in Scheme 2. The key intermediate **DTTDO-I** was prepared in the Horner-Wadsworth-Emmons olefination reaction of bisphosphonate **5** with aldehyde **7**. Next, the transformation of four terminal iodide groups in **DTTDO-I** into the corresponding quaternary ammonium salt **DTTDO-Py^+^** was achieved by treatment of **DTTDO-I** with an excess of pyridine. **DTTDO-MOR** was obtained in the nucleophilic substitution reaction of **DTTDO-I** with morpholine. A similar reaction of **DTTDO-I** with bis(2-hydroxyethyl)amine afforded **DTTDO-DEA** in 88% yield.

### 2.2. Optical Properties of DTTDO Derivatives

**Figure 4:**
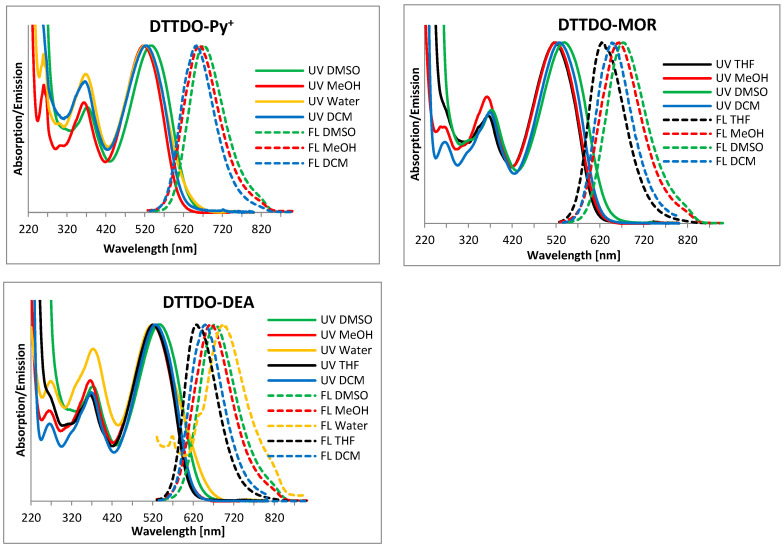
Normalized fluorescence (dotted line) and UV-VIS (solid line) spectra of **DTTDO** derivatives in different solvents.

To determine the optical properties of the **DTTDO** derivatives their UV-VIS and fluorescence spectra in five solvents of different polarity (water, DMSO, MeOH, THF, and DCM) were recorded. Stokes shift (∆λ), fluorescence quantum yield (Ф), as well as absorption (λ_abs_) and emission (λ_em_) maxima of these compounds, are shown in Table 3 while Figure 4 presents their normalized fluorescence and UV-VIS spectra.

Due to the presence of quaternary ammonium and hydroxyl groups in **DTTDO-Py^+^**, and **DTTDO-DEA,** respectively, these compounds are well soluble in water, DMSO, DCM, and MeOH but only **DTTDO-DEA** turned out to be also soluble in THF. Although it was possible to obtain the fluorescence spectrum of **DTTDO-Py^+^** in THF, the broad and low-intensity absorption spectrum did not allow us to reliably determine its maxima. On the other hand, **DTTDO-MOR** which has four terminal morpholinyl groups is insoluble in H_2_O but well soluble in other organic solvents. All investigated compounds have λ_abs_ maxima in the range of 517–538 nm.

Both water-soluble compounds do not exhibit (**DTTDO-Py^+^**) or exhibit (**DTTDO-DEA**) only a very weak fluorescence in water but their fluorescence is easily detected when dissolved in organic solvents. Because pendant groups attached to the six-carbon atom alkyl chain do not participate in the π-electron delocalization, the optical properties of **DTTDO** derivatives are largely independent of their type. For a given solvent, the observed difference between λ_abs_ or λ_em_ maxima is only in the range of a few nanometers.

**Figure 5:**
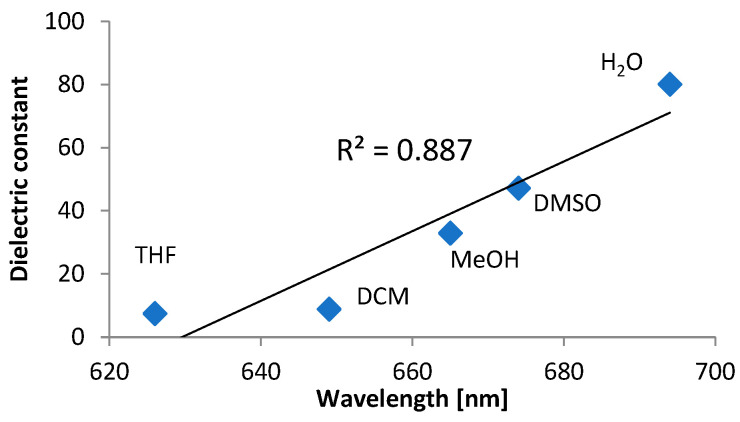
Correlation between maximum emission wavelength of **DTTDO-DEA** and solvent dielectric constant.

As all of the investigated compounds have a permanent dipole moment due to the presence of a strong electron-withdrawing sulphonyl group, it could be expected that absorption and emission spectra could be affected by the solvent polarity. While in the case of UV-VIS spectra such a relationship was not seen, for emission spectra a bathochromic shift of *λ*_em_ to a longer wavelength was observed with an increase of the solvent dielectric constant (Figure 5).

It indicates that the exited state of **DTTDO** derivatives is more polarized than the ground state and polar solvent molecules better stabilize orbitals involved in the electronic transition. The highest λ_em_ maximum (694 nm) and the larger Stokes shift (174 nm) were observed for **DTTDO-DEA** in water.

Also, the fluorescence quantum yield of **DTTDO** derivatives strongly depends on the solvent polarity. While in DMSO the Ф values are low and do not exceed a few percent, they are significantly higher (about 3 times) in less polar dichloromethane. Because **DTTDO** derivatives are not soluble in a solvent of a low polarity it was not possible to determine their Ф in e.g., hexane or cyclohexane which would better mimic properties of the plasma membrane. The highest fluorescence quantum yield of 19.5% was registered for **DTTDO-DEA** in DCM.

### 2.3. Detection of Fluorescent Signal Inside the Cells

**Figure 6:**
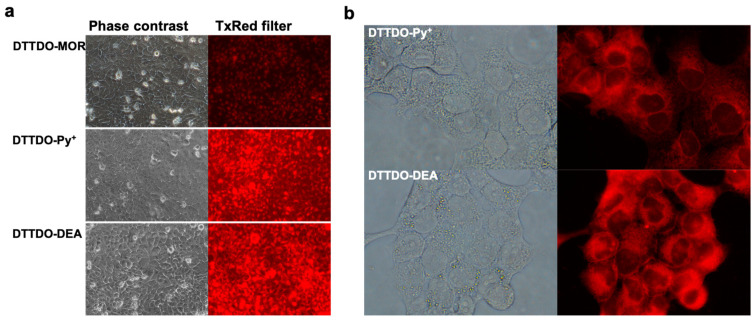
Intercellular fluorescence of **DTTDO** derivatives in living human keratinocytes (**a**) and squamous carcinoma cells (**b**) after incubation under standard cell culture conditions in a complete medium containing FBS and antibiotics. Compounds concentration 10 µM, incubation time—24 h. Left panel—phase contrast, right panel—fluorescent images with TxRed filter (λ_ex_ = 540–580 nm, λ_DM_ = 595 nm, λ_BA_ = 600–660 nm). Magnification and exposure time: 10×, 600 ms for (**a**) and 60×, 2 s for (**b**), respectively.

To determine the applicability and selectivity of **DTTDO** derivatives to cell imaging their staining properties under different cellular conditions were investigated. Microscopic observation revealed that analyzed compounds penetrated living cells and effectively stained selected cellular structures. Furthermore, no modification in the standard cell culture conditions containing antibiotics and Fetal Bovine Serum was required to observe a fluorescent signal, making it possible to use these compounds for real-time microscopic observations (Figure 6).

**Figure 7:**
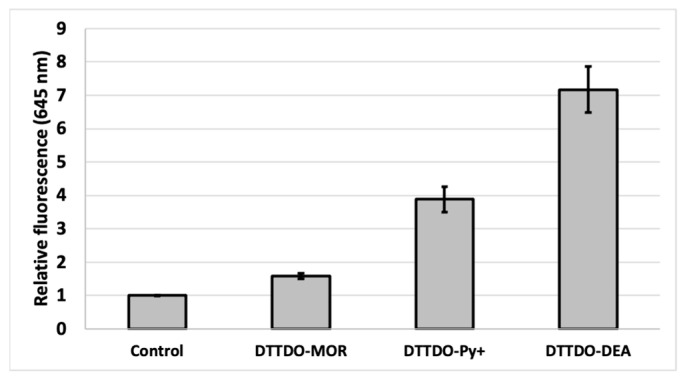
The intensity of fluorescence in A431 cells after 1 h treatment with **DTTDO** derivatives. Results represent the mean fluorescent value at *λ*_em_ = 645/40 nm with *λ*_ex_ = 530/25 nm calculated to control cells (cells treated with DMSO). Compounds concentration 10 µM.

While the obtained results showed efficient staining of cell structures with an inconsiderable medium background for all tested **DTTDO** derivatives, the intensity of fluorescent signal inside the cells differed depending on the compound. Among all tested dyes, the lowest fluorescence intensity was observed for **DTTDO-MOR**. Both the remaining compounds demonstrated strong labeling of cells. To quantify the intracellular fluorescence signal, additional fluorescence intensity measurements were performed. For this purpose, the compounds were added to cell cultures and incubated in standard conditions for one hour in a complete medium. Before the measurements, the cells were washed twice to eliminate the background error and ensure, that obtained fluorescence value represented the signal from the cells. The obtained data confirmed the results from microscopy observations, indicating **DTTDO-MOR** as the least effective among the tested dyes, and **DTTDO-DEA** as the most efficient one (Figure 7). Furthermore, the results revealed, that a one-hour incubation is sufficient to observe the fluorescent signal from all the tested compounds inside living cells.

### 2.4. Cytotoxicity

For an assessment of the possibility to use **DTTDO** derivatives for staining purposes in living cells their impact on cell viability in the concentration range that allows effective cell staining was estimated. To evaluate the cytotoxic properties of the tested dyes, two types of model cells: cancer and non-cancerous human cells were selected. Cancer cells were represented by epidermoid squamous carcinoma cells (A431), while human keratinocytes were used as the model of normal cells.

**Figure 8:**
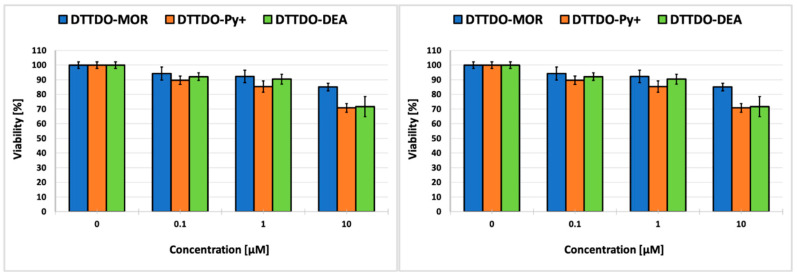
Cytotoxicity of tested compounds for human epidermoid squamous carcinoma cells (A431) (**left** panel) and human keratinocytes (**right** panel) after 24 h incubation. Results represent the mean cell viability calculated with reference control (cells treated with DMSO—100% viability).

In the concentration range used (0.1–10 μM), no significant toxic effect was observed after a 24-h incubation of the cells with the compounds both in the case of A431 cancer cells and in non-cancerous keratinocytes. The cell survival rate remained above 70% for all tested compounds (Figure 8). At a concentration enabling efficient cell staining, none of the tested compounds exhibited cytotoxicity after 24 h of incubation. The above results indicate that these compounds can be safely used for living cell staining.

## 3. Materials and Methods

### 3.1. Synthesis of DTTDO Derivatives

#### 3.1.1. General Remarks

All water- and air-sensitive reactions were conducted in the neutral gas atmosphere using freshly distilled dry solvents. Dry THF and DCM were prepared by distillation over Na/benzophenone and calcium hydride, respectively. Commercial-grade reagents and solvents were used without further purification except as indicated above. NMR spectra were recorded on Bruker AV Neo 400 (Bruker, Billerica, MA, USA) or Avance III 600 (Bruker) spectrometers. ^1^H, ^13^C, and ^31^P chemical shifts are reported relative to the residual proton resonance in the deuterated solvents or referred to 85% phosphoric acid, respectively. All chemical shifts (δ) are given in ppm. HRMS measurements were performed on a Finnigan MAT 95 (Thermo Fisher Scientific, Waltham, MA, USA) or Waters Synapt HDMS (Waters Corporation, Milford, MA, USA) mass spectrometer. Melting points are uncorrected. 4-(*N*,*N*-bis(6-iodohexyl)amino)benzaldehyde (**7**) was prepared according to the literature procedure [41]. NMR spectra of all synthesized compounds are available in Supplementary Materials.

#### 3.1.2. Synthetic Procedures

**2,6**-**Bis(hydroxymethyl)dithieno[3,2-*b*:2′,3′-d]thiophene (2):** To a suspension of LiAlH_4_ (0.49 g, 12.8 mmol) in THF (10 mL) at 0 °C a suspension of 2,6-diethyl dithieno [3,2-*b*:2′,3′-*d*]thiophene dicarboxylate (**1**) (1.75 g, 5.1 mmol) in THF (10 mL) was added in several portions. The reaction mixture was stirred at room temperature for 1 h, and AcOEt (10 mL) followed by water (20 mL) was added carefully. The mixture was extracted with AcOEt (3 × 30 mL) and the combined organic layers were dried over anhydrous magnesium sulfate. Evaporation of the solvent under reduced pressure afforded the crude diol **2** (1.31 g, quant.) as a white solid, which was used in the next step without purification. Mp 156–158 °C; ^1^H NMR (400 MHz, DMSO-*d*_6_) δ 7.31 (s, 2H, C*H*), 5.99–5.39 (br s, 2H, O*H*), 4.68 (s, 4H, C*H*_2_); ^13^C NMR (126 MHz, DMSO-*d*_6_) δ 147.65 (*C*CH_2_), 139.50 (*C*H), 129.30, 118.17, 58.99 (*C*H_2_); Anal. calcd. for C_10_H_8_O_2_S_3_ (256.35): C, 46.85; H, 3.15; S, 37.52. Found: C, 46.72; H, 3.12; S, 37.33.

**2,6-Bis(dichloromethyl)dithieno[3,2-*b*:2′,3′-*d*]thiophene (3):** Thionyl chloride (1.78 g, 15 mmol) was added to a suspension of diol **2** (1.28 g, 5 mmol) in DCM (20 mL) and a mixture was stirred at room temperature for 4 h. The reaction mixture was carefully poured into a saturated ice-cold NaHCO_3_ aqueous solution and extracted with DCM (3 × 30 mL). The combined organic layers were dried over anhydrous magnesium sulfate. Filtration followed by concentration under reduced pressure yielded **3** (1.40 g, 96%) as a greyish solid which was used in the next reaction without further purification. Mp 190–192 °C (decomposition); ^1^H NMR (400 MHz, CDCl_3_) δ 7.29 (s, 2H, C*H*), 4.88 (s, 4H, C*H*_2_Cl); ^13^C NMR (126 MHz, CDCl_3_) δ 141.39, 140.65 (*C*H), 131.79, 121.41 (*C*CH_2_), 41.48 (*C*H_2_); HRMS(EI) *m*/*z*: [M]^+^ calcd. for C_10_H_6_Cl_2_S_3_ 291.9009, found 291.9023; Anal. calcd. for C_10_H_8_Cl_2_S_3_ (293.24): C, 40.96; H, 2.06; S, 32.80. Found: C, 40.78; H, 2.30; S, 32.70.

**2,6-Bis[(diethoxyphosphoryl)methyl]dithieno[3,2-*b*:2′,3′-*d*]thiophene (4):** Dichloride **3** (1.40 g, 4.8 mmol) and triethyl phosphate (9.96 g, 60 mmol, 10.3 mL) were heated at 150 °C for 24 h. An excess of triethyl phosphate was removed under reduced pressure and the residue was purified by column chromatography (diethyl ether/acetone 5:1) affording **4** (2.26 g, 95%) as a yellow solid. Mp 80–82 °C; *R*_f_ = 0.2 (diethyl ether/acetone 5:1); ^31^P NMR (81 MHz, CDCl_3_) δ 23.48; ^1^H NMR (400 MHz, CDCl_3_) δ 7.17 (d, *J*_HP_ = 3.7 Hz, 2H, C*H*), 4.10 (quint, 8H, POC*H*_2_), 3.42 (d, *J*_HP_ = 21.2 Hz, 4H, PC*H*_2_), 1.30 (t, *J*_HH_ = 7.1 Hz, 12H, C*H*_3_); ^13^C NMR (400 MHz, CDCl_3_) δ 140.19, 133.39 (d, *J*_CP_ = 10.3 Hz, *C*-CH_2_), 130.24, 120.96 (d, *J*_CP_ = 9.1 Hz, *C*H), 62.73 (d, *J*_CP_ = 7.0 Hz, PO*C*H_2_), 29.32 (d, *J*_CP_ = 143.3 Hz, *C*H_2_P), 16.58 (d, *J*_CP_ = 6.0 Hz, *C*H_3_); HRMS(CI) *m*/*z*: [M+H]^+^ calcd. for C_18_H_27_O_6_P_2_S_3_ 497.0446, found 497.0445.

**2,6-Bis[(diethoxyphosphoryl)methyl]dithieno[3,2-*b*:2′,3′-*d*]thiophene-4,4-dioxide (5):** To a solution of bisphosphonate **4** (2.26 g, 4.6 mmol) in CH_2_Cl_2_ (20 mL), *m*-CPBA (2.78 g, 16.1 mmol) was added and a mixture was stirred at room temperature for 24 h. A saturated aqueous solution of NaHCO_3_ (20 mL) was added and the mixture was extracted with CH_2_Cl_2_ (3 × 20 mL). The combined organic layers were washed with H_2_O (20 mL) and dried over anhydrous magnesium sulfate. After evaporation of the solvent, the residue was purified by column chromatography (CH_2_Cl_2_/acetone 10:1) affording dioxide **5** (2.33 g, 97%) as a yellow solid. Mp 116–118 °C; *R*_f_ = 0.42 (CH_2_Cl_2_/acetone 2:1); ^31^P NMR (81 MHz, CDCl_3_): δ 22.12; ^1^H NMR (101 MHz, CDCl_3_) δ 7.04 (d, *J*_HP_ = 3.4 Hz, 2H, C*H*), 4.16–4.09 (m, 8H, POC*H*_2_), 3.34 (d, *J*_HP_ = 21.1 Hz, 4H, C*H*_2_), 1.32 (t, *J*_HH_ = 7.1 Hz, 12H, C*H*_3_); ^13^C NMR (400 MHz, CDCl_3_) δ 141.73, 138.67 (d, *J*_CP_ = 11.1 Hz, *C*CH_2_), 135.21, 119.96 (d, *J*_CP_ = 9.7 Hz, *C*H), 63.00 (d, *J*_CP_ = 7.1 Hz, PO*C*H_2_), 29.12 (d, *J*_CP_ = 144.2 Hz, *C*H_2_P), 16.58 (d, *J*_CP_ = 6.3 Hz, *C*H_3_); HRMS(CI) *m*/*z*: [M+H]^+^ calcd. for C_18_H_27_O_8_P_2_S_3_ 529.0343, found 529.0337.

**6-Bis[(diethoxyphosphoryl)methyl]dithieno[3,2-*b*:2′,3′-*d*]thiophene-4-oxide (6):** When oxidation of bisphosphonate **4** was carried out according to the above procedure but using a smaller excess of *m*-CPBA (2.5 eq.), next to 4,4-dioxide **5**, sulphoxide **6** was isolated as a yellow solid in 26% yield. Mp 82–84 °C; *R*_f_ = 0.13 (CH_2_Cl_2_/acetone 2:1); ^31^P NMR (81 MHz, CDCl_3_) δ 22.59; ^1^H NMR (400 MHz, CDCl_3_) δ 7.22 (d, *J*_HP_ = 3.4 Hz, 2H, C*H*), 4.16–4.08 (m, 8H, OC*H*_2_), 3.36 (d, *J*_HP_ = 21.0 Hz, 4H, PC*H*_2_), 1.32 (td, *J*_HP_ = 7.1, *J*_HH_ = 1.5 Hz, 12H, C*H*_3_); ^13^C NMR (101 MHz, CDCl_3_): δ 148.76, 137.81, 137.01 (d, *J*_CP_ = 11.1 Hz, *C*CH_2_), 123.67 (d, *J*_CP_ = 9.6 Hz, *C*H), 62.90 (O*C*H_2_), 29.11 (d, *J*_CP_ = 144.3 Hz, *C*H_2_P), 16.60 (d, *J*_CP_ = 6.2 Hz, *C*H_3_); HRMS(ES+) *m*/*z*: [M+H]^+^ calcd. for C_18_H_27_O_7_P_2_S_3_ 513.0394, found 513.0407.

**2,6-Bis{4-[*N*,*N*-bis(6-iodohexyl)amino]styryl}dithieno[3,2-*b*:2′,3′-*d*]thiophene-4,4-dioxide (DTTDO-I):** Bisphosphonate **5** (0.39 g, 0.74 mmol) and aldehyde **7** (0.84 g, 1.55 mmol) were dissolved in THF (60 mL) at 0 °C. Sodium *tert*-butoxide (1.10 mL 2M in THF, 2.21 mmol) was added and the cooling bath was removed. After stirring for 4.5 h the reaction mixture was quenched with a 5% HCl solution and extracted with CHCl_3_ (3 × 50 mL). The combined organic layers were dried over anhydrous MgSO_4_. After evaporation of the solvents under reduced pressure, the remaining material was purified by column chromatography (petroleum ether/ethyl acetate 10:1) to give **DTTDO-I** (0.70 g, 77%) as a dark red solid. Mp 249–250 °C; *R*_f_ = 0.45 (ethyl acetate); ^1^H NMR (600 MHz, CDCl_3_) δ 7.31 (d, *J* = 8.6 Hz, 4H, C_Ph_*H*), 6.99 (s, 2H, C_DTTDO_*H*), 6.87 (d, *J* = 16.0 Hz, 2H, C*H*=CH), 6.84 (d, *J* = 16.0 Hz, 2H, C*H*=CH), 6.59 (d, *J* = 8.5 Hz, 4H, C_Ph_*H*), 3.29 (t, *J* = 7.6 Hz, 8H, NC*H*_2_), 3.20 (t, *J* = 6.9 Hz, 8H, C*H*_2_I), 1.84 (quint, *J* = 7.1 Hz, 8H, C*H*_2_CH_2_I), 1.61 (quint, *J* = 7.6 Hz, 8H, NCH_2_C*H*_2_), 1.46 (quint, *J* = 7.4 Hz, 8H, C*H*_2_CH_2_CH_2_I), 1.36 (quint, *J* = 7.5 Hz, 8H, NCH_2_CH_2_C*H*_2_); ^13^C NMR (151 MHz, CDCl_3_) δ 149.28 (2C, *C*_DTTDO_), 148.41 (2C, N*C*_Ph_), 142.51 (2C, *C*_DTTDO_), 132.30 (2C, *C*_DTTDO_), 131.05 (2C, *C*H=CH), 128.37 (4C, H*C*_Ph_), 123.24 (2C, CH*C*_Ph_), 115.93 (2C, H*C*_DTTDO_), 115.68 (2C, *C*H=CH), 111.85 (4C, H*C*_Ph_), 51.02 (4 C, N*C*H_2_), 33.52 (4C, CH_2_*C*H_2_CH_2_), 30.46 (4C, CH_2_*C*H_2_CH_2_), 27.27 (4C, CH_2_*C*H_2_CH_2_), 26.18 (4C, CH_2_*C*H_2_CH_2_), 7.09 (4C, *C*H_2_I); HRMS (ES+) *m*/*z*: [M+H]^+^ calcd for C_48_H_63_I_4_N_2_O_2_S_3_ 1303.1235; found 1303.1234; Anal. calcd. for C_48_H_62_I_4_N_2_O_2_S_3_ (1302.83): C, 44.25; H, 4.80; S, 7.38; N, 2.15. Found: C, 44.35; H, 4.68; S, 7.20.

**2,6-Bis(4-{*N*,*N*-bis[6-(pyridinium)hexyl]amino}styryl)dithieno[3,2-*b*:2′,3′-*d*]thiophene-4,4-dioxide (DTTDO-Py^+^).** A solution of tetraiodide **DTTDO-I** (50 mg, 0.038 mmol) and pyridine (1 mL) in methanol (4 mL) was stirred at 45 °C for 8 h. The solvent and pyridine were evaporated under reduced pressure and the residue was dissolved in methanol. Precipitation with diethyl ether afforded **DTTDO-Py^+^** (60 mg, 96%) as a dark purple solid. Mp 125–6 °C; ^1^H NMR (400 MHz, DMSO-*d*_6_) δ 9.08 (d, *J* = 5.4 Hz, 8H, *H(2)_Py_*), 8.61 (tt, *J* = 7.7, 1.4 Hz, 4H, *H(4)_Py_*), 8.17 (dd, *J* = 7.8, 6.5 Hz, 8H, *H(3)_Py_*), 7.47 (s, 2H, *H*C*_DTTDO_*), 7.37 (d, *J* = 8.5 Hz, 4H, C_Ph_*H*), 7.12 (d, *J* = 16.0 Hz, 2H, C*H*=CH), 7.00 (d, *J* = 16.0 Hz, 2H, C*H*=CH), 6.62 (d, *J* = 8.5 Hz, 4H, C_Ph_*H*), 4.59 (t, *J* = 7.5 Hz, 8H, C*H*_2_N^+^), 3.31 (br s, 8H, C*H*_2_N), 1.92 (quint, *J* = 7.1 Hz, 8H, C*H*_2_CH_2_N^+^), 1.58–1.24 (m, 24H, C*H*_2_). ^13^C NMR (101 MHz, DMSO) δ 149.47 (2C, *C_DTTDO_*), 147.94 (2C, *C*_Ph_N), 145.48(4C, *C(4)_Py_*), 144.68 (8C, *C(2)_Py_*), 141.93 (2C, *C_DTTDO_*), 131.25 (2C, *C_DTTDO_*), 130.79 (2C, *C*H=CH), 128.34 (4C, *C*_Ph_H), 128.07(8C, *C(3)_Py_*), 122.51 (2C, *C*_Ar_), 115.80 (2C, H*C_DTTDO_*), 115.28 (2C, *C*H=CH), 111.44 (4C, *C*_Ph_H), 60.62 (4C, *C*H_2_N^+^), 49.88 (4C, *C*H_2_N), 30.69 (4C, *C*H_2_CH_2_N^+^), 26.61 (4C, *C*H_2_), 25.77 (4C, *C*H_2_), 25.33 (4C, *C*H_2_); HRMS (ES+) *m*/*z*: [M-I]^+^ calcd for C_68_H_82_I_3_N_6_O_2_S_3_ 1491.2799; found 1491.2795; Anal. calcd. for C_68_H_82_I_4_N_6_O_2_S_3_ (1619.24): C, 50.44; H, 5.10; S, 5.94. Found: C, 50.32; H, 5.12; S, 6.12.

**2,6-Bis(4-{*N*,*N*-bis[6-(4-morpholinyl)hexyl]amino}styryl)dithieno[3,2-*b*:2′,3′-*d*]thiophene-4,4-dioxide (DTTDO-MOR): DTTDO-I** (58 mg, 0.045 mmol) and morpholine (0.387 mg, 4.45 mmol) were dissolved in THF (12 mL) and heated at 70 °C for 8 h. Volatile ingredients were removed under reduced pressure, and a saturated aqueous solution of NaHCO_3_ (5 mL) was added. The mixture was extracted with DCM (3 × 30 mL). The combined organic extracts were washed with water (5 mL) and dried over Na_2_SO_4_. Solvent evaporation followed by the purification of the crude material by column chromatography (DCM/MOH = 10:1.5) afforded **DTTDO-MOR** (47 mg, 93%) as a dark purple solid. Mp 53–5 °C; *R*_f_ = 0.3 (DCM/MeOH = 10:1.5); ^1^H NMR (400 MHz, CDCl_3_) δ 7.57 (d, *J* = 8.3 Hz, 4H, C_Ph_*H*), 7.52 (s, 2H, C_DTTDO_*H*), 7.14 (d, *J* = 16.2 Hz, 2H, C*H*=CH), 7.10 (d, *J* = 15.8 Hz, 2H, C*H*=CH), 6.85 (d, *J* = 8.5 Hz, 4H, C_Ph_*H*), 3.99 (t, *J* = 4.7 Hz, 16H, OC*H*_2_), 3.55 (t, *J* = 7.6 Hz, 8H, C_Ph_NC*H*_2_), 2.70 (br s, 16H, NC*H*_2_CH_2_O), 2.60 (t, *J* = 7.6 Hz, 8H, OCH_2_CH_2_NC*H*_2_), 1.90–1.72 (m, 16H, C*H*_2_), 1.65–1.56 (m, 16H, C*H*_2_); ^13^C NMR (101 MHz, CDCl_3_) δ 149.26 (2C, *C_DTTDO_*), 148.41 (2C, *C*_Ph_N), 142.43 (2C, *C*_DTTDO_), 132.26 (2C, *C*_DTTDO_), 131.03 (2C, *C*H=CH), 128.31 (4C, *C*_Ph_H), 123.00, 115.89 (2C, *C*_DTTDO_H), 115.55 (2C, *C*H=CH), 111.72 (4C, *C*_Ph_H), 67.06 (8C, O*C*H_2_), 59.19 (4C, OCH_2_CH_2_N*C*H_2_), 53.89 (8C, N*C*H_2_CH_2_O), 51.10 (4C, C_Ph_N*C*H_2_), 27.53 (4C, *C*H_2_), 27.39 (4C, *C*H_2_), 27.20 (4C, *C*H_2_), 26.66 (4C, *C*H_2_); HRMS (ES+) *m*/*z*: [M+H]^+^ calcd for C_64_H_95_N_6_O_2_S_3_ 1139.6483; found 1139.6475; Anal. calcd. For C_64_H_94_N_6_O_6_S_3_ (1139.67): C, 67.45; H, 8.31; S, 8.44. Found: C, 67.66; H, 8.35; S, 8.30.

**2,6-Bis[4-(*N*,*N*-bis{6-[bis(2-hydroxyethyl)amino]hexyl}amino)styryl]dithieno[3,2-*b*:2′,3′-*d*]thiophene-4,4-dioxide (DTTDO-DEA):** A solution of **DTTDO-I** (53 mg, 0.041 mmol) and diethanolamine (0.35 g, 3.33 mmol) in THF (12 mL) was heated at 70 °C for 6 h. Volatile ingredients were evaporated under reduced pressure. Then, saturated aqueous NaHCO_3_ solution (5 mL) was added and the mixture was extracted with CH_2_Cl_2_ (3 × 20 mL). The combined organic extracts were washed with water and dried over Na_2_SO_4_. Solvent evaporation followed by the purification of the crude material by column chromatography (DCM/MeOH/Et_3_N = 5:15:0.4) afforded **DTTDO-DEA** (43 mg, 88%) as a dark purple solid. Mp 76–78 °C; ^1^H NMR (400 MHz, CD_3_OD) δ 7.13 (d, *J* = 7.9 Hz, 4H, C_Ph_*H*), 6.89 (s, 2H, C_DTTDO_*H*), 6.62 (br s, 4H, CH=C*H*), 6.48 (d, *J* = 7.9 Hz, 4H, C_Ph_*H*), 3.59 (t, *J* = 5.9 Hz, 16H, OC*H*_2_), 3.16 (br s, 8H), 2.62 (t, *J* = 5.9 Hz, 16H, NC*H*_2_CH_2_O), 2.51 (t, *J* = 7.2 Hz, 8H, C_Ph_NC*H*_2_), 1.55–1.39 (m, 16H, C*H*_2_), 1.32–1.22 (m, 16H, C*H*_2_); ^13^C NMR (101 MHz, CD_3_OD) δ 151.03, 149.63, 143.38, 133.25, 131.93 (2C, *C*H=CH), 129.61 (4C, *C*_Ph_H), 124.43, 116.75 (2C, *C*_DTTDO_H), 116.33 (2C, *C*H=CH), 112.81 (4C, *C*_Ph_H), 60.69 (8C, O*C*H_2_), 57.58 (8C, N*C*H_2_CH_2_O), 56.24 (4C, OCH_2_CH_2_N*C*H_2_), 51.95 (4C, C_Ph_N*C*H_2_), 28.65 (4C, *C*H_2_), 28.55 (4C, *C*H_2_), 28.23 (4C, *C*H_2_), 27.88 (4C, *C*H_2_); HRMS (ES+) *m*/*z*: [M+H]^+^ calcd for C_64_H_103_N_6_O_10_S_3_ 1211.6890; found 1211.6898; Anal. calcd. for C_64_H_102_N_6_O_10_S_3_ (1211.73): C, 63.44; H, 8.49; S, 6.94. Found: C, 63.71; H, 8.40; S, 6.81.

### 3.2. Computational Methods

Geometries of all compounds were optimized without symmetry restrictions at the Second Order Møller-Plesset perturbation theory (MP2) [49] using the frozen-core approximation and triple-zeta Dunning’s correlation-consistent cc-pVTZ basis set [50] (atomic coordinates of **DTT**, **DTTMO,** and **DTTDO** are available in Supplementary Materials). Stationary points found on the potential energy surface were confirmed to be equilibrium structures by calculation of vibrational frequencies at the same level as the geometry optimization. These optimized structures were subsequently used for single-point calculations of molecular properties. All DFT and wave-function-based calculations were performed using Gaussian 09 D.01 [51]. “Ultrafine” integration grid and tight convergence criteria were applied in all cases. Nucleus-independent chemical shifts (NICS) [47] and harmonic oscillator model of aromaticity (HOMA) [48] index were employed for the analyses of the local aromaticity. The HOMA index was calculated according to the following formula

(1) $$\mathrm{HOMA}=1-\frac{1}{N}\sum_{i}^{N}\alpha_{i,j}{\left(R_{opt}-R_{i,j}\right)}^{2}$$

where *N* is the number of bonds in the considered ring, $\alpha_{i,j}$ is constant for a specific pair of connected atoms, *R_opt_* is an optimal bond length for a fully delocalized π-electron reference system, and *R_i_*_,*j*_ is the actual bond length between *i* and *j* atoms. For fully aromatic rings HOMA is equal 1, which means that each bond length is equal to the optimal value *R_opt_*. Pre-calculated constants proposed by Krygowski [48] were taken for HOMA index calculations (CC bond: α_CC_ = 257.7, *R_opt_* = 1.388; CS bond: α_CS_ = 94.09, *R_opt_* = 1.677). The NICS index, which is defined as the opposite value of magnetic shielding at the specified location in space, was calculated at the mp2/cc-pVTZ geometries using m06L [52] functional and cc-pVTZ basis set. The negative NICS value indicates the presence of an induced diatropic ring current (aromatic character) whereas the positive one specifies a paratropic ring current and denotes antiaromaticity. Following the recommendation of Schleyer and co-workers [47], in our aromaticity analysis, the NICS variant denoted as NICS(1)zz was applied and the absolute magnetic shielding was calculated 1 Å above the ring critical point. To predict favorable reactive sites and reactivity character dual descriptor ∆f(r) for nucleophilicity and electrophilicity was calculated [46]. This index is defined as the difference between nucleophilic and electrophilic Fukui functions [53] and is expressed by the following formula:

(2) $$∆f\left(r\right)=f^{+}\left(r\right)-f^{-}\left(r\right)$$

Numerical values of ∆f(r) are defined within the range {−1; 1}.The positive value indicates that the site is favored for the nucleophilic attack, whereas the negative one suggests that the site is more susceptible to the reaction with electrophiles. Dual descriptors ∆f(r) were calculated using the PBE0 [54] functional and aug-cc-pVTZ [55,56] basis set. Calculation of the HOMA aromaticity index and local reactivity dual descriptors was conducted using Multiwfn 3.8 software [57].

### 3.3. Optical Characterization

The absorption and emission spectra were recorded on Shimadzu UV-VIS spectrophotometer UV 2700 (Shimadzu Scientific, Kyoto, Japan) and Horriba Scientific FluoroMax+ spectrofluorimeter (Horriba Scientific, Kyoto, Japan), respectively, at room temperature. All measurements were conducted using spectrophotometric grade solvents. Fluorescence spectra were recorded after excitation at maximum absorption wavelength. Fluorescence quantum yield (Ф) was calculated using Rhodamine 101 in EtOH as a reference (Ф = 1).

### 3.4. Cell Culture Conditions

The biological part of the research was performed using two types of human cells: cancer—squamous carcinoma cells (A431) and non-cancerous—human keratinocytes. The human keratinocytes came from Leibniz Institute DSMZ-German Collection of Microorganisms and Cell Cultures (Braunschweig, Germany). The A431 cells were purchased from American Type Culture Collection (ATCC, Manassas, Virginia, USA). Both types of cells were cultured in Dulbecco’s modified Eagle’s medium (DMEM) (Sigma-Aldrich, St. Louis, MO, USA) supplemented with 10% fetal bovine serum (FBS) (Sigma-Aldrich, St. Louis, MO, USA) and 100 U/mL penicillin and 100 μg/mL streptomycin (Invitrogen). Cells were grown at 37 °C under a 5% CO_2_ atmosphere. For each experiment, cells were counted using ScepterTM 2.0 Cell Counter (Merck, Darmstadt, Germany).

### 3.5. Measurements of Cellular Fluorescence Intensity

The A431 cells were seeded into 96-well black plates for fluorescent measurements at a concentration of 10,000 cells per well and then allowed to adhere for 24 h in the cell culture incubator under standard conditions (37 °C and 5% CO_2_). After that time, the culture medium from each well was removed and the new medium containing tested compounds at a concentration of 10 μM was added. Cells were incubated with tested dyes for 1 h under standard cell culture conditions (37 °C and 5% CO_2_). After treatment, the medium containing **DTTDO** derivatives was removed and cells were washed twice with PBS buffer to avoid a possible background signal. After washing, cell fluorescence was measured by Synergy HT microplate reader (Bio-Tek, Winooski, VT, USA) at an excitation and emission wavelength range of *λ*_ex_ = 530/25 and *λ*_em_ = 645/40 nm, respectively.

The cells treated only with DMSO were used as a control. The mean fluorescence value of these cells was used as a reference. The fluorescence value for DTTDO-treated cells was calculated related to the control sample. Each experiment was performed thrice in three repetitions.

### 3.6. Cell Viability Test

The cytotoxicity of compounds was assessed using the MTT test, based on the measurement of the activity of mitochondrial dehydrogenase, which converts yellow tetrazolium salts ((3-(4,5-dimethyl-2-thiazolyl)-2,5-diphenyltetrazolium bromide) into water-insoluble formazan. The tests were performed based on the previously described procedure [58]. The cells were seeded into 96-well plates at a concentration of 10,000 cells per well and left under standard conditions (37 °C, 5% CO_2_) for 24 h. Then, the culture medium was removed and a fresh medium containing DMSO (negative control), SDS (positive control), or test compounds dissolved in DMSO was added. After a 24-h incubation, the (3-(4,5-dimethyl-2-thiazolyl)-2,5-diphenyltetrazolium bromide was added to the culture medium. After a 2-h incubation at 37 °C, the medium was removed, and 100 µL of isopropanol was added to each well and shaken for an hour at room temperature. The absorbance was measured at 570 nm (evaluation of formazan amount) and 630 nm (reference value) using a Synergy HT plate reader (Bio-Tek) with KC4 3.2 Rev 2 software (Bio-Tek). The compounds were tested at concentrations of 0.1, 1, and 10 µM. The final amount of DMSO in each well was 1%. Each variant was repeated at least three times in each experiment. The cytotoxicity level of each compound was calculated relative to the negative control (DMSO-treated cells) set at 100%. Each viability point represents the mean value from at least three independent experiments performed thrice.

### 3.7. Visualization of Compounds Inside the Cell

For the fluorescence microscopy imaging 50,000 cells were seeded into each well of a 24-well plate and incubated in standard conditions (37 °C, 5% CO_2_) for 24 h. Then, the medium was removed and a new medium containing DTTDO derivatives at the final concentration of 10 μM was added. Cells were then cultured with tested compounds in a cell culture incubator under standard conditions (37 °C, 5% CO_2_). At the 24 h time point, the fluorescence of the cells was observed using a Nis Eclipse Ti microscope (Nikon, Tokyo, Japan). The microscopic observations were performed immediately on the plates without changing the cell culture medium. Cells were observed using a TxRed filter (λ_ex_ = 540–580 nm, λ_DM_ = 595 nm, λ_BA_ = 600–660 nm; DM is a dichroic mirror and BA is an absorption filter). Data were recorded and analyzed using Nis-Elements BR 4.30.00 software (Nikon).

## 4. Conclusions

In summary, the simple and effective synthesis of a series of fluorescent 2,6-divinyl **DTTDO** derivatives was accomplished in six steps starting from 2,6-diethyl dithienothiophene dicarboxylate in 66–60% overall yield. The key steps of the synthesis involved the preparation of tetraiodide **DTTDO-I** in the Horner-Wadsworth-Emmons reaction of bisphosphonate **5** with aldehyde **7**, followed by its conversion into the appropriate **DTTDO** derivatives in the reaction with different nucleophiles. The synthesized compounds turned out to be selective fluorescent dyes for staining membranous structures of the cells. They exhibit emission in the red light for which autofluorescence from the biological samples is highly reduced. Moreover, their fluorogenic properties and negligible cytotoxicity effects at the concentration required for effective staining enable *real-time* observations of living cells under standard cell culture conditions in a whole cell medium containing serum and antibiotics. Additionally, **DTTDO** dyes are also suitable for multicolor experiments with compounds that exhibit green or blue fluorescence.

## Figures and Tables

**Table 1:** Optical properties of unoxidized and oxidized bisphosphonates **4**–**6** recorded in dichloromethane.

Compound	λ_abs_ [nm]	λ_em_ [nm]	Stokes Shift [nm]
4	319	353	34
5	371	462	91
6	379	489	109

**Table 2:** Calculated aromaticity indexes, dual descriptors, and energy of frontier orbitals of **DTT**, **DTTMO,** and **DTTDO**.

Compound	HOMA ^a^	NICS(1)zz ^b^	∆f(r) ^c^ S(4)	E_HOMO_ ^c^ [eV]	E_LUMO_ ^c^ [eV]	E_LUMO—_E_HOMO_ ^c^ [eV]
**DTT**	A: 0.864 B: 0.788	A: −22.6 B: −17.8	−0.133	−6.04	−1.28	4.76
**DTTMO**	A: 0.919 B: 0.322	A: −21.2 (−21.6) ^d^ B: 6.5 (5.3) ^d^	0.041	−6.39	−2.13	4.26
**DTTDO**	A: 0.932 B: 0.417	A: −21.0 B: 12.1	0.019	−6.60	−2.35	4.25

^a^ Calculated at the mp2/cc-pVTZ geometry; ^b^ Calculated at the m06L/cc-pVTZ//mp2/cc-pVTZ level; ^c^ Calculated at the pbe0/aug-cc-pVTZ//mp2/cc-pVTZ level; ^d^ Due to the non-planar geometry of the middle thiophene ring, NICS(1)zz values at the two sides of the ring are not equivalent.

**Table 3:** Maximum absorption (λ_abs_), emission (λ_em_)**,** Stokes shift (∆λ), and quantum yield (Ф) of **DTTDO** derivatives in different solvents ^a^.

Compound	H_2_O		MeOH		DMSO			THF		DCM		
	λ_abs_/λ_em_	∆λ	λ_abs_/λ_em_	∆λ	λ_abs_/λ_em_	∆λ	Ф	λ_abs_/λ_em_	∆λ	λ_abs_/λ_em_	∆λ	Ф
**DTTDO-Py^+^**	520/-	-	517/661	144	536/672	136	5.6	nd ^c^/622	-	523/653	130	16.1
**DTTDO-MOR**	ns ^b^/-	-	519/664	145	538/674	136	6.0	517/626	109	527/647	120	17.0
**DTTDO-DEA**	520/694	174	520/665	145	536/674	138	6.0	520/626	106	526/649	123	19.5

^a^ λ_abs_, λ_em_, ∆λ values are given in [nm] and Ф in [%]; ^b^ ns—not soluble; ^c^ nd—not determined because of low solubility.

## Data Availability

The data presented in this study are available in Supplementary Materials.

## Supplementary Materials

The following supporting information can be downloaded at: https://www.mdpi.com/article/10.3390/ma16051806/s1: absorption and emission spectra of bisphosphonates **4**–**6**; atomic coordinates of **DTT**, **DTTMO,** and **DTTDO** optimized at the mp2/cc-pVTZ level; NMR spectra of all synthesized compounds.
