# Supplementary material for: Red-Emitting Dithienothiophene S,S-Dioxide Dyes for Cellular Membrane Staining
Source: Materials (Basel). 2023 Feb 22;16(5):1806. doi: 10.3390/ma16051806 (PMC10003865; doi:10.3390/ma16051806)
Supplement: Supplementary file 1 [file materials-16-01806-s001.zip › materials-2192622-supplementary.pdf]

Supporting Information

Red-Emitting Dithienothiophene *S,S*-Dioxide Dyes for Cellular Membrane Staining

Aneta Rzewnicka [1], Jerzy Krysiak [1], Róża Pawłowska [2,*] and Remigiusz Żurawiński [1,*]

[1] Division of Organic Chemistry, Centre of Molecular and Macromolecular Studies, Polish Academy of Sciences, Sienkiewicza 112, 90-363 Lodz, Poland
[2] Division of Bioorganic Chemistry, Centre of Molecular and Macromolecular Studies, Polish Academy of Sciences, Sienkiewicza 112, 90-363 Lodz, Poland
* Correspondence: roza.pawlowska@cbmm.lodz.pl (R.P.); remigiusz.zurawinski@cbmm.lodz.pl (R.Ż.)

TABLE OF CONTENTS

1. Absorption and emission spectra S2
2. Atomic coordinates S3
3. NMR spectra S4

1. Absorption of an Emission Spectra

Normalized absorption (blue) and emission (red) spectra of bisphosphonates **4**, **5**, and **6** in DCM

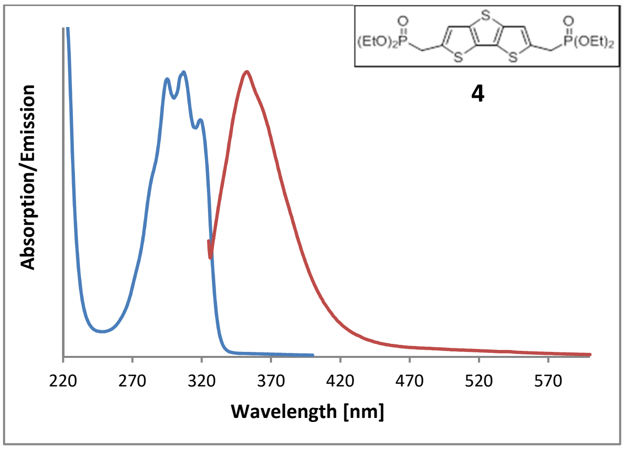

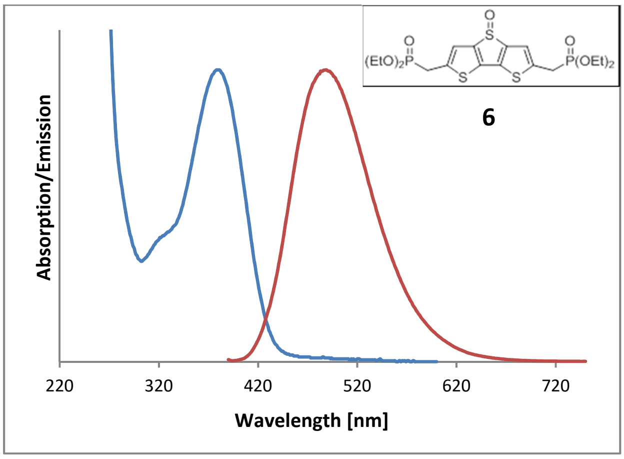

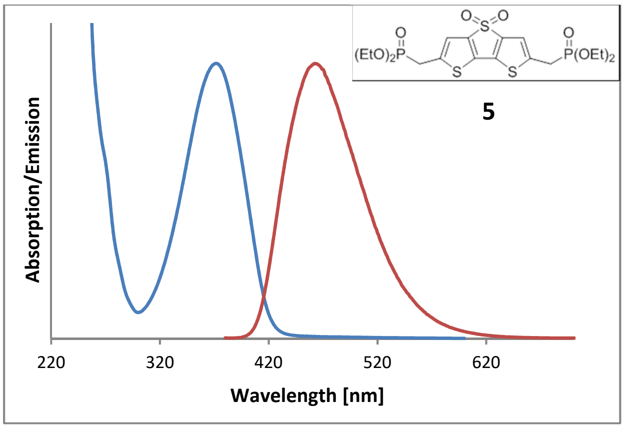

2. Atomic coordinates of DTT, DTTMO, and DTTDO optimized at the mp2/cc-pVTZ level

DTT

C,-0.0010446696,-0.0000000009,0.0029786539|C ,-0.0006034832,0.0000000004,1.4162114056|C,1.2869611504,0.0000000024,1.974573708|S,2.4891529741,0.0000000031,0.7468792205|C,1.2823274088,0.0000000004,-0.4908418709|S,-1.2516988953,0.0000000005,2.6266255757|C,-0.0542429379,0.0000000005,3.8901296708|C,1.2563130836,-0.0000000017,3.388099861|C,-0.1159386848,0.0000000007,5.3020149707|C,1.1448233697,-0.0000000007,5.8509976651|S,2.4041622323,-0.0000000029,4.6667478355|H,-1.0204143254,0.0000000019,5.8905829314|H,-0.8791597417,-0.0000000028,-0.6242392801|H,1.588116781,-0.0000000003,-1.5242489533|H,1.405533738,-0.0000000006,6.8966876061|

DTTMO

C,-0.0040327482,-0.0017210013,0.0033092759|C,-0.0009098935,-0.0087213432,1.4090313745|C,1.2728796545,-0.0091028872,1.9669909107|S,2.4762704864,0.0163547875,0.7546868324|C,1.2841813145,0.0267731908,-0.4917614296|C,-0.0488929711,-0.0029307115,3.910824417|C,1.2453545173,-0.0057811638,3.402125972|C,-0.1059148269,0.010574379,5.3153455073|C,1.1623710562,0.0414739881,5.8593110633|S,2.40137723,0.0253927415,4.6595512958|H,-1.0092140466,0.0205008434,5.9058029864|H,-0.8840258882,0.0053927171,-0.6213902669|S,-1.3033893051,-0.1963799125,2.6358482259|O,-2.2655287881,0.9461842639,2.6147503324|H,1.5986661602,0.0587473095,-1.5220707457|H,1.4371206086,0.0782438687,6.9007624586|

DTTDO

C,0.,0.,0.|C,0.,0.,1.40268526|C,1.26400029,0.,1.97370377|S,2.47310899,0.,0.7692661|C,1.29353093,0.,-0.48528196|C,-0.03300328,0.,3.95496789|C,1.24533888,0.,3.41682512|C,-0.06927426,0.,5.35718512|C,1.21127562,0.,5.87575224|S,2.42289864,0.,4.65212547|H,-0.96218949,0.,5.96255716|H,-0.87696279,0.,-0.62825983|S,-1.262023,0.,2.66272038|O,-1.98866734,1.25492295,2.65332384|O,-1.98866618,-1.25492305,2.65332506|H,1.61529408,0.,-1.51374648|H,1.5063369,0.,6.91219308|

3. NMR spectra

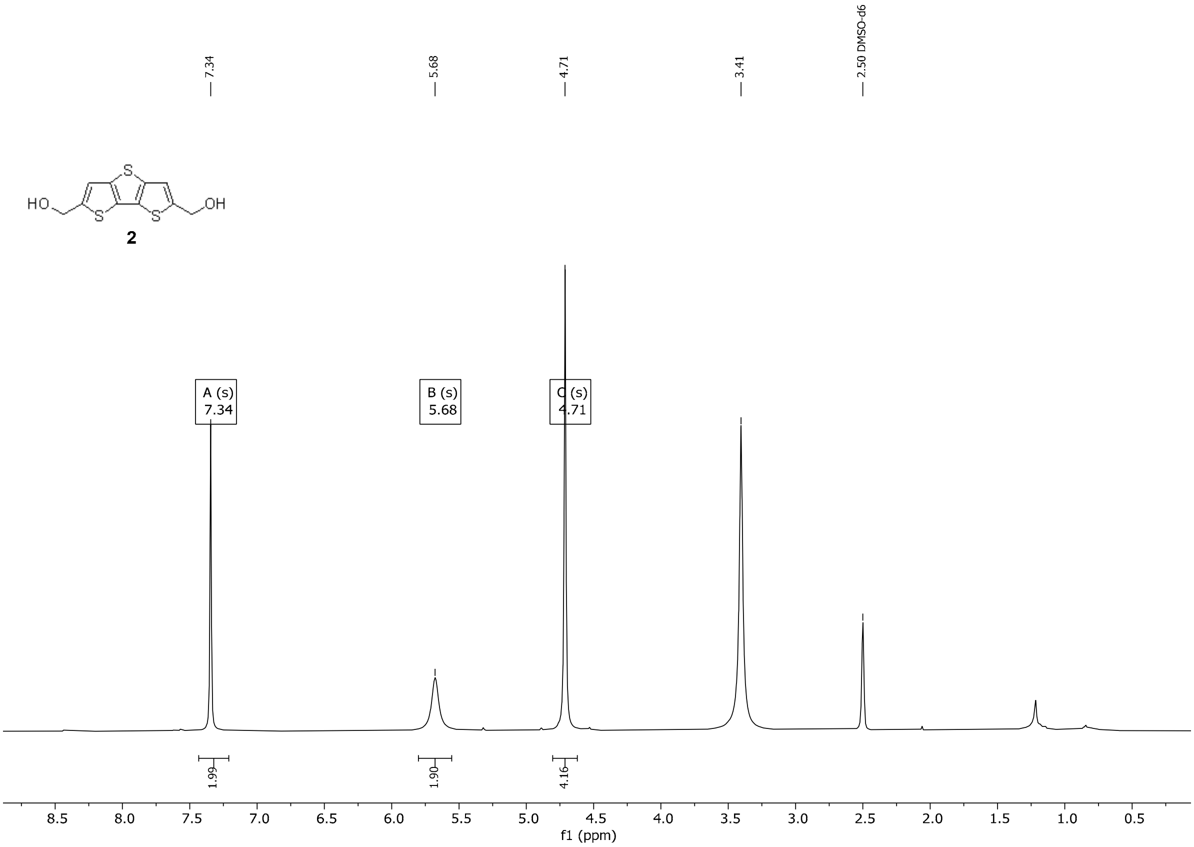

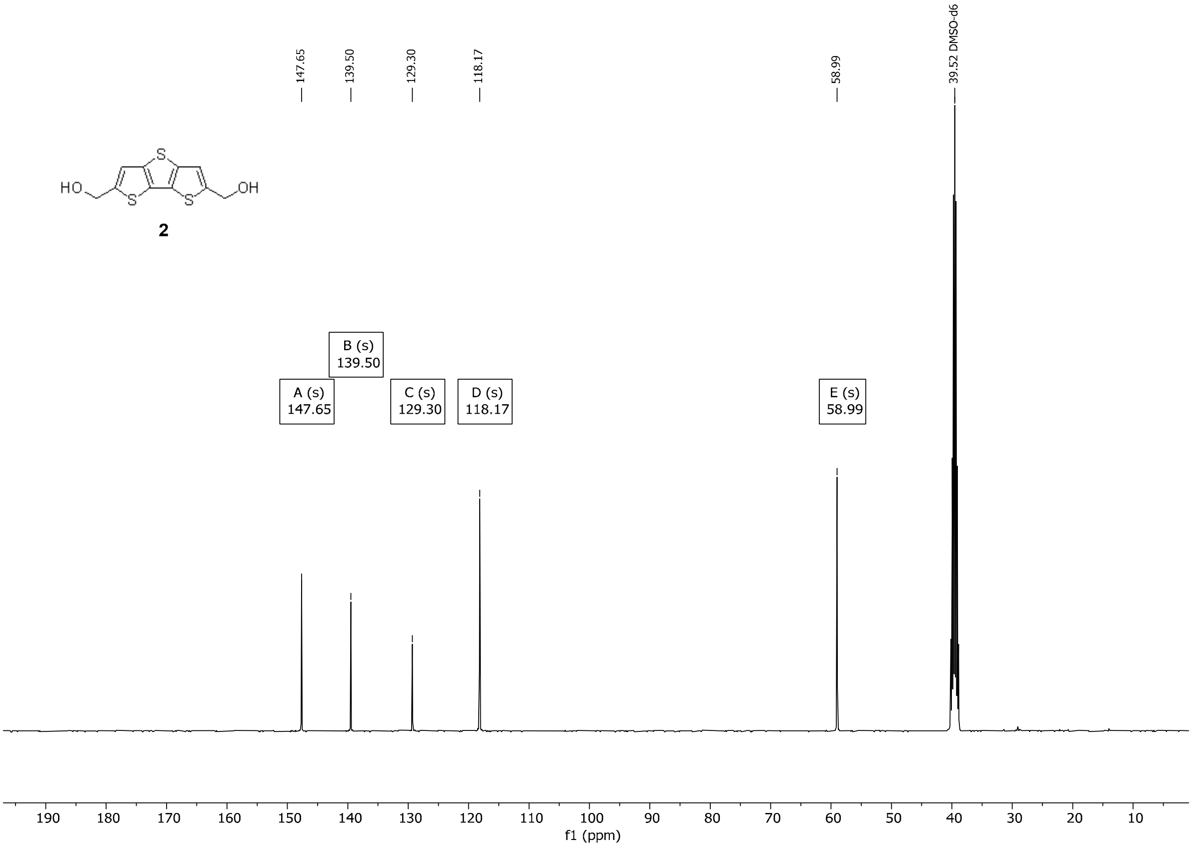

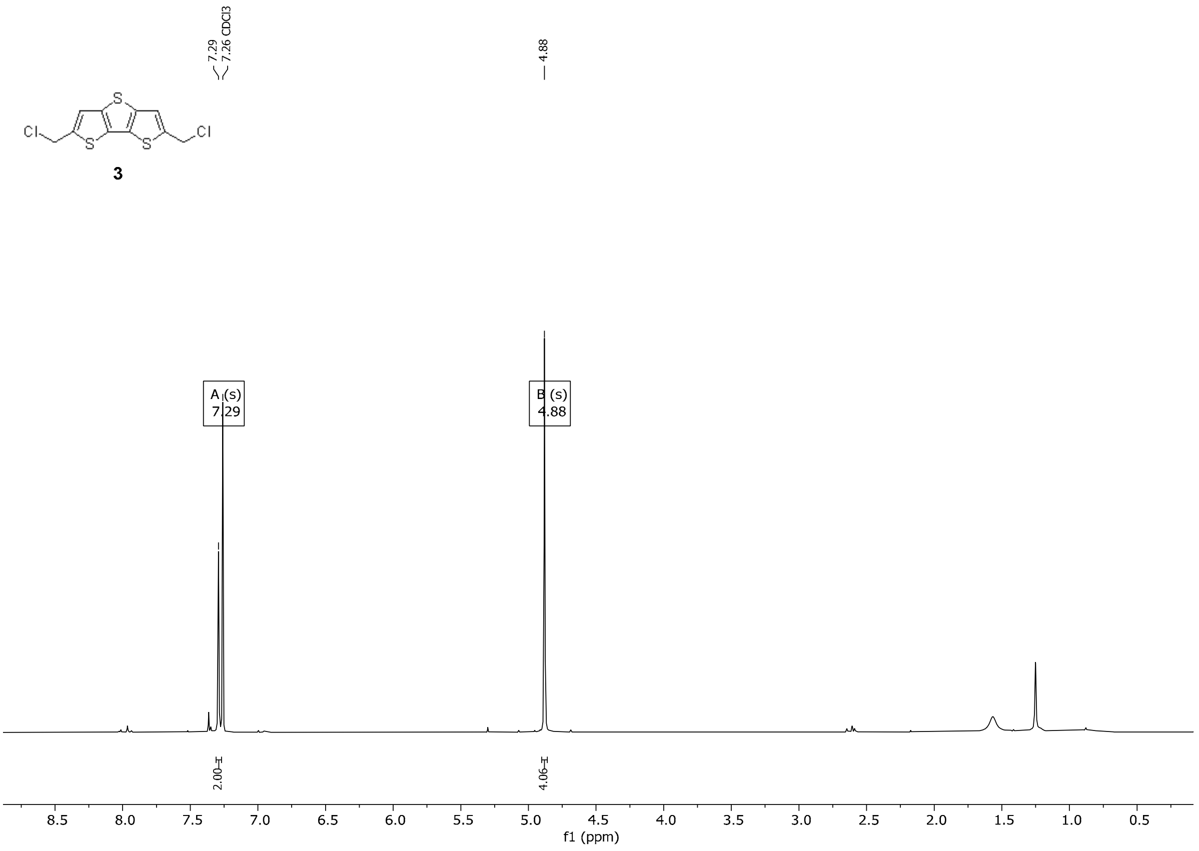
7.29
7.26 CDCl3
4.88
3
A (s)
7.29
B (s)
4.88
2.00
4.06
f1 (ppm)

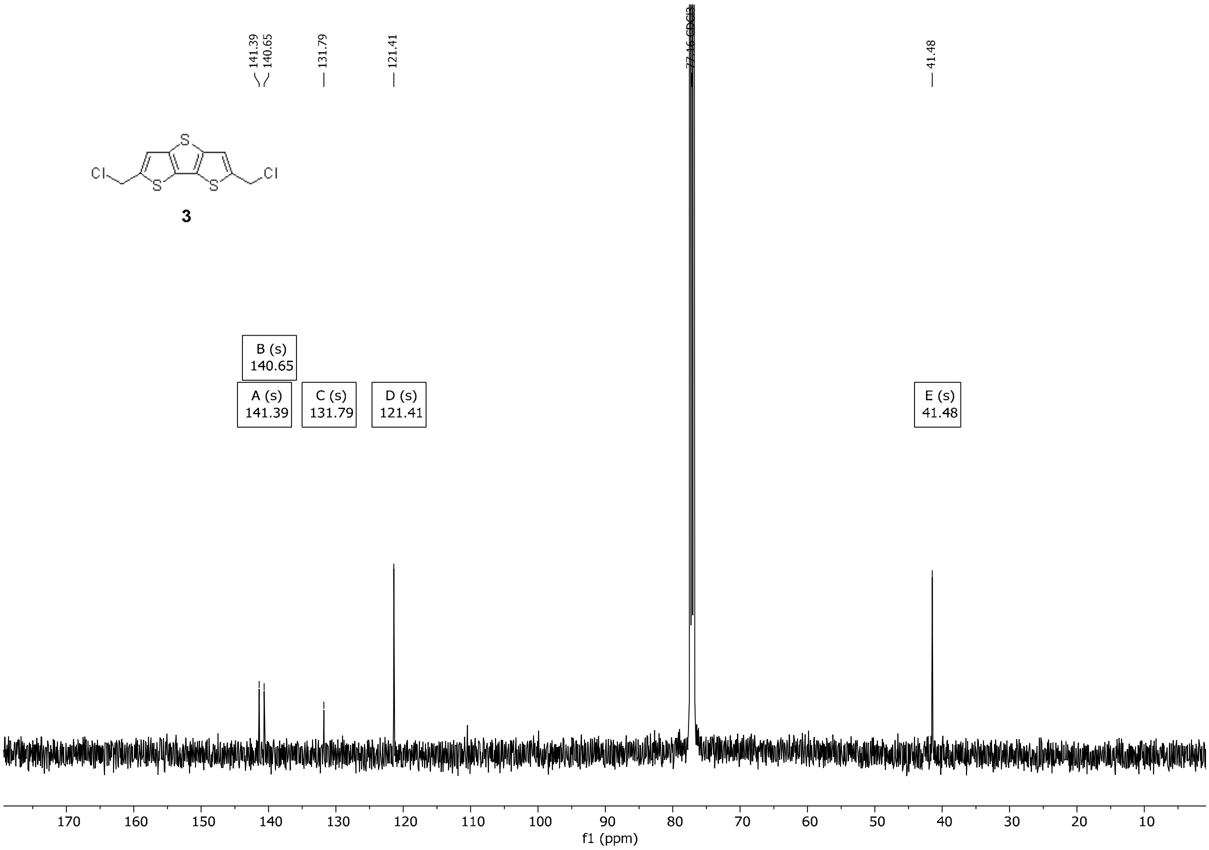
141.39
140.65
131.79
121.41
77.16 CDCl3
41.48
3
B (s)
140.65
A (s)
141.39
C (s)
131.79
D (s)
121.41
E (s)
41.48
f1 (ppm)

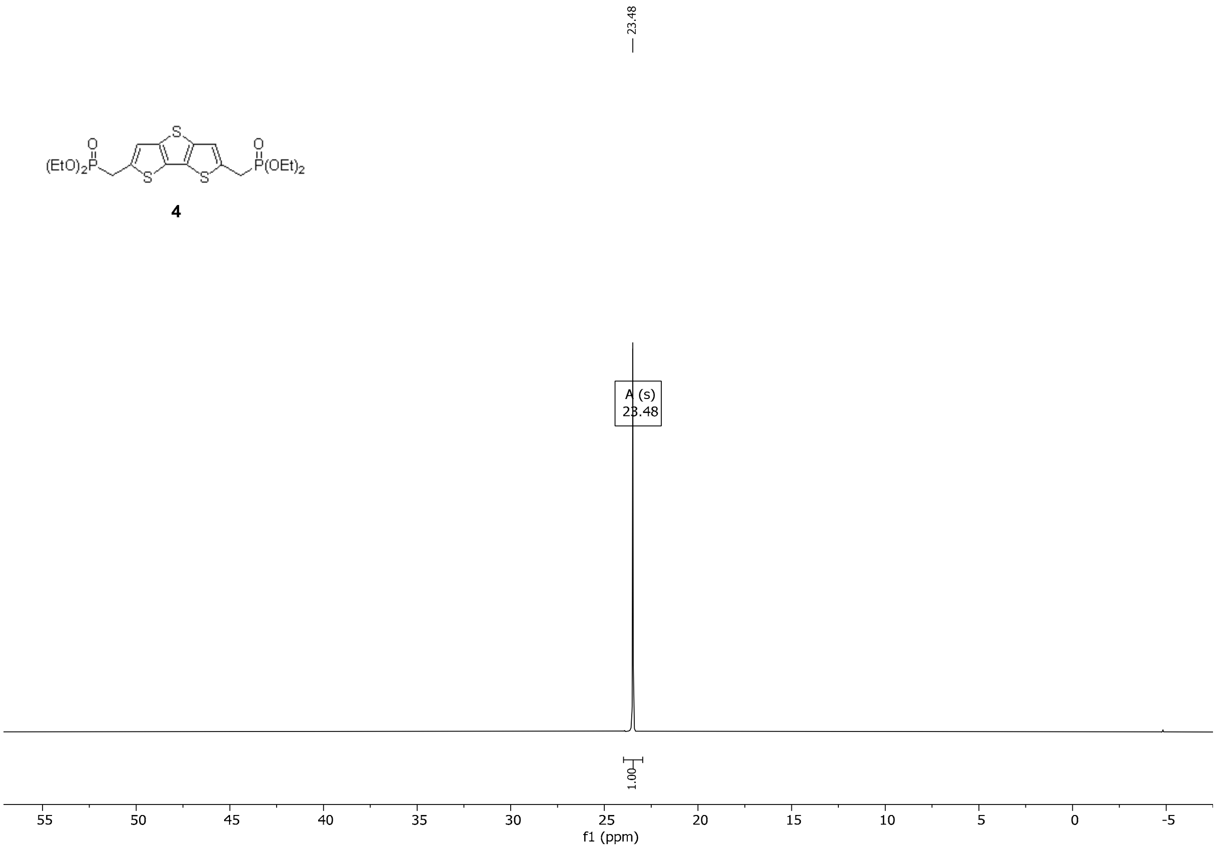

23.48
(EtO)2P(O)
P(OEt)2
4
A (s)
23.48
1.00
55 50 45 40 35 30 25 20 15 10 5 0 -5
f1 (ppm)

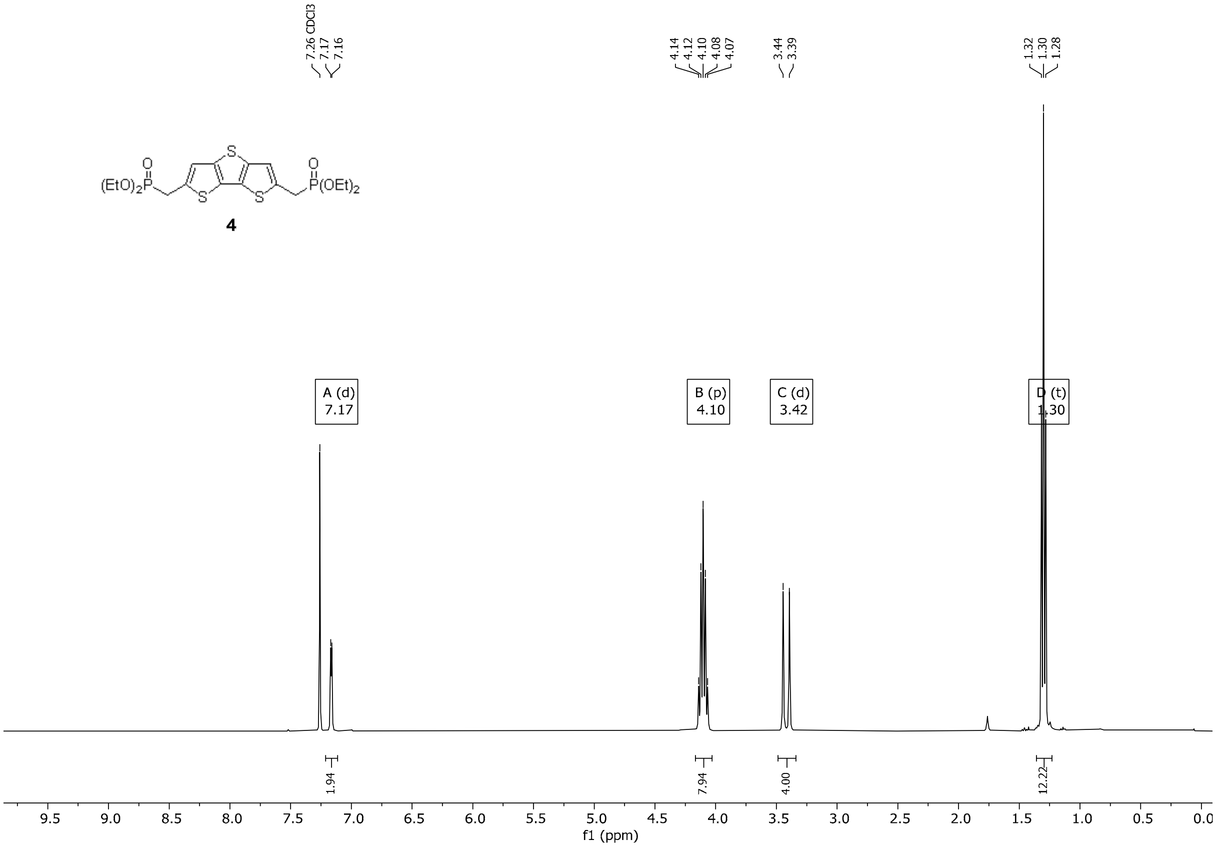

7.26 CDCl3
7.17
7.16
4.14
4.12
4.10
4.08
4.07
3.44
3.39
1.32
1.30
1.28
(EtO)2P(O)
P(OEt)2
4
A (d)
7.17
B (p)
4.10
C (d)
3.42
D (t)
1.30
1.94
7.94
4.00
12.22
9.5 9.0 8.5 8.0 7.5 7.0 6.5 6.0 5.5 5.0 4.5 4.0 3.5 3.0 2.5 2.0 1.5 1.0 0.5 0.0
f1 (ppm)

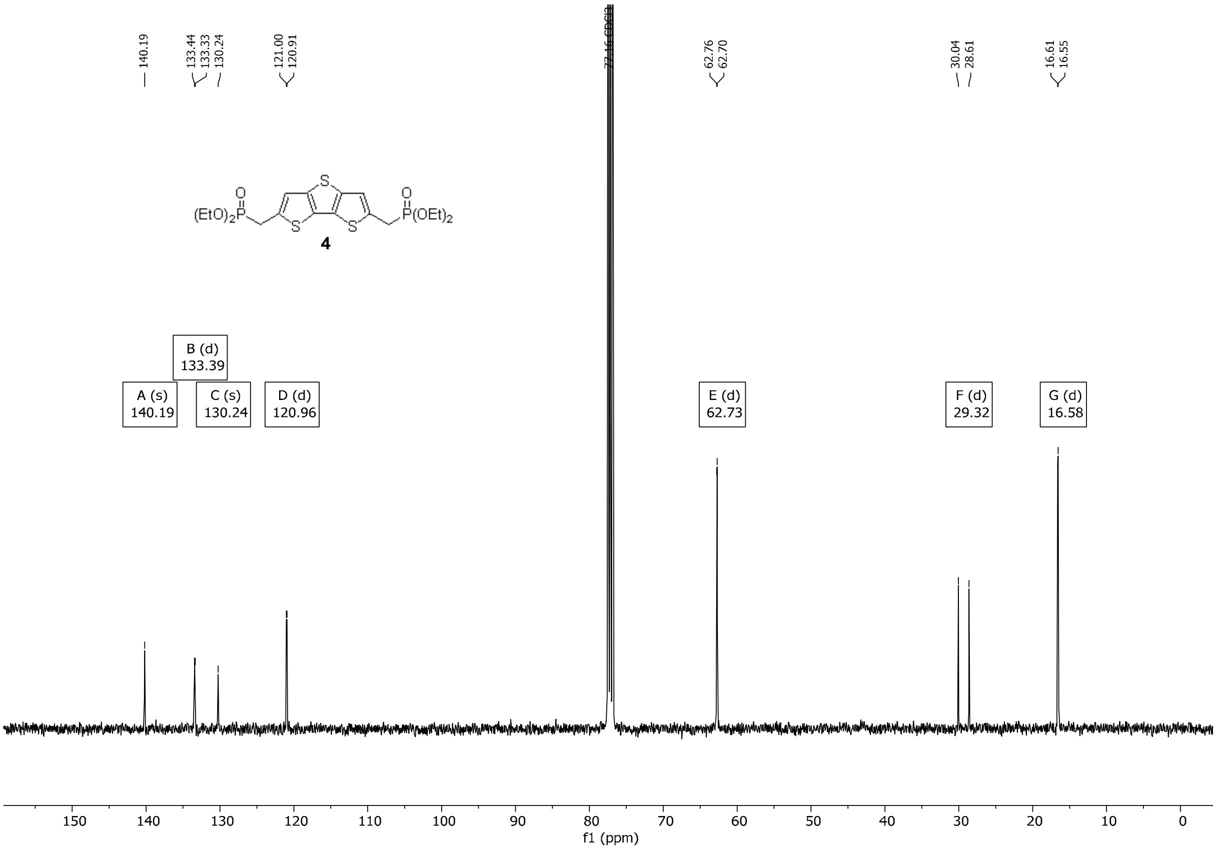
140.19
133.44
133.33
130.24
121.00
120.91
77.16 CDCl3
62.76
62.70
30.04
28.61
16.61
16.55
(EtO)2P
P(OEt)2
4
B (d)
133.39
A (s)
140.19
C (s)
130.24
D (d)
120.96
E (d)
62.73
F (d)
29.32
G (d)
16.58
150 140 130 120 110 100 90 80 70 60 50 40 30 20 10 0
f1 (ppm)

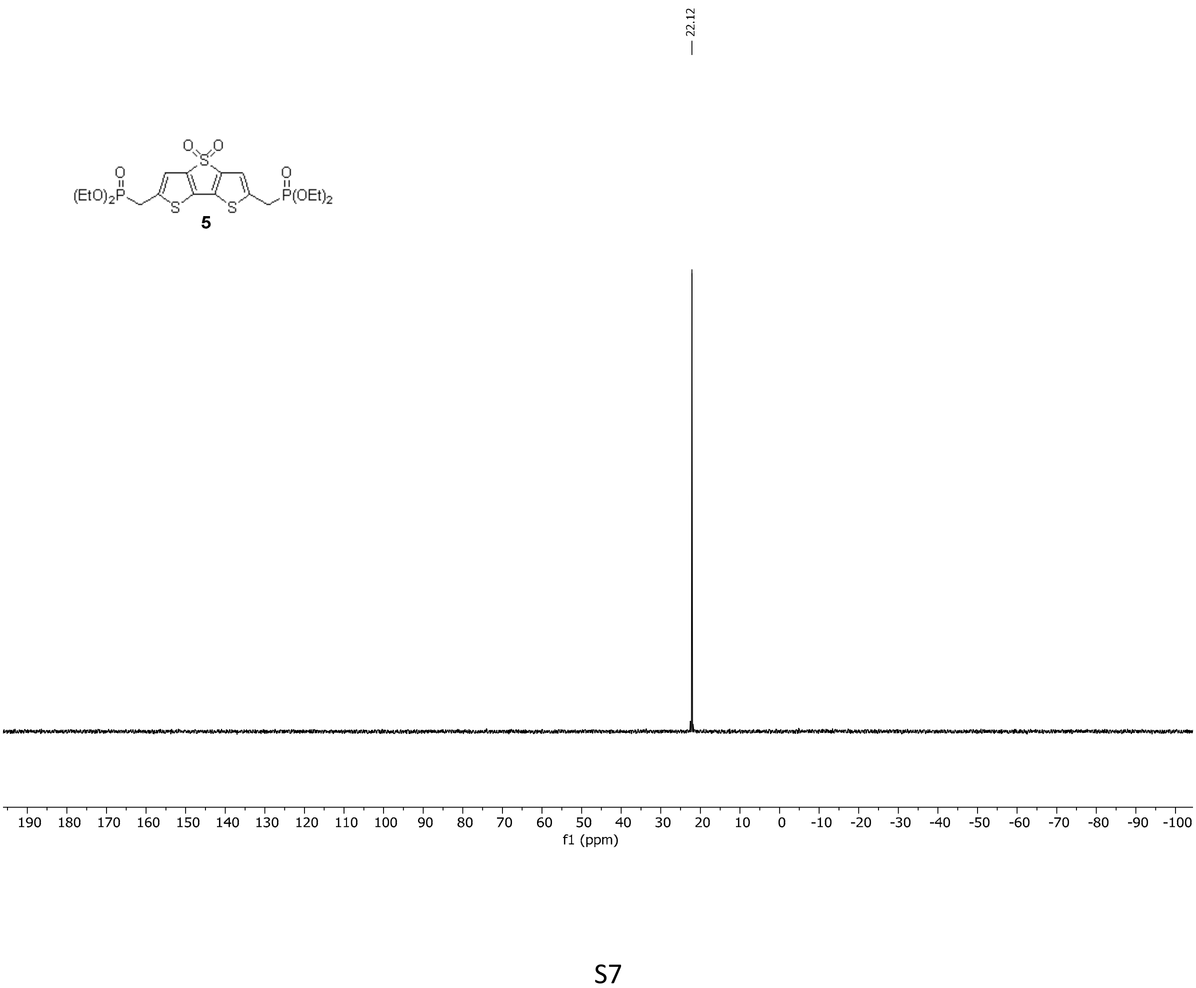
22.12
(EtO)2P
P(OEt)2
5
190 180 170 160 150 140 130 120 110 100 90 80 70 60 50 40 30 20 10 0 -10 -20 -30 -40 -50 -60 -70 -80 -90 -100
f1 (ppm)

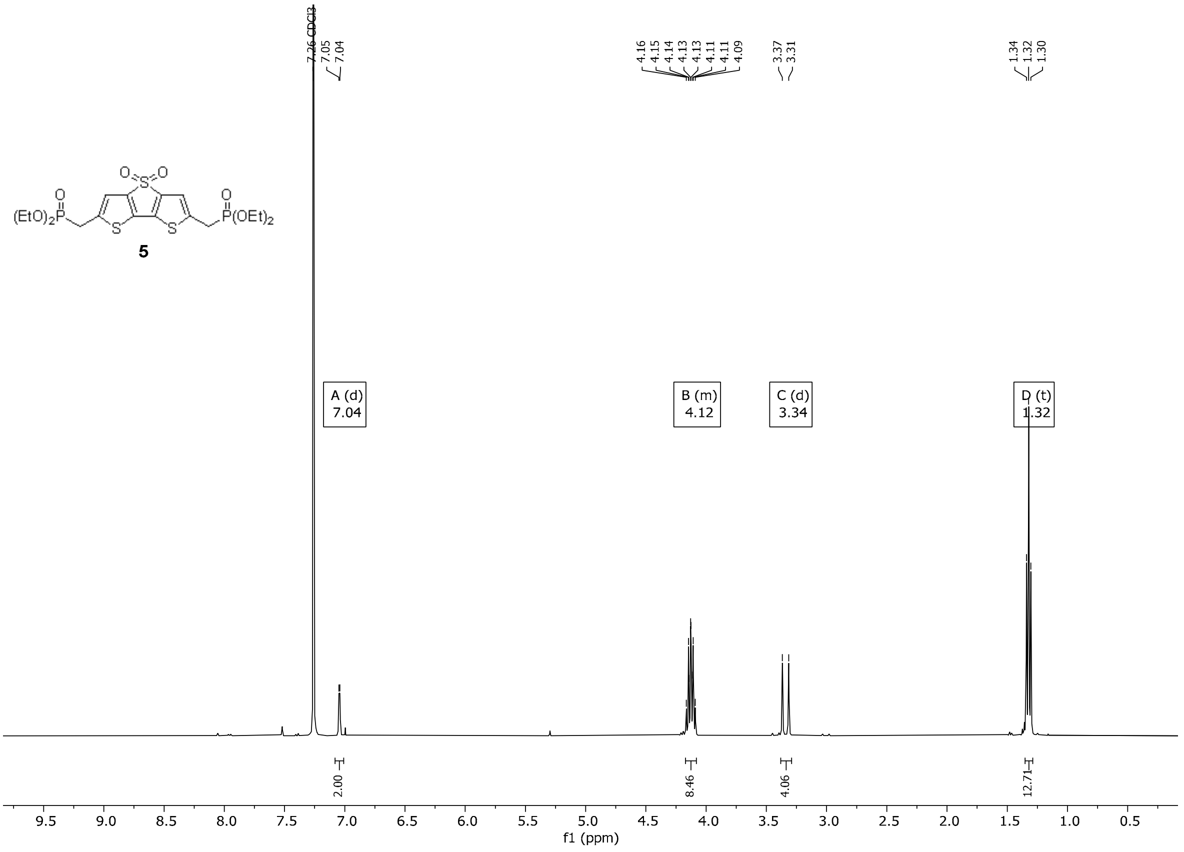
7.26 CDCl3
7.05
7.04
4.16
4.15
4.14
4.13
4.13
4.13
4.11
4.11
4.09
3.37
3.31
1.34
1.32
1.30
(EtO)2P
P(OEt)2
5
A (d)
7.04
B (m)
4.12
C (d)
3.34
D (t)
1.32
2.00
8.46
4.06
12.71
f1 (ppm)

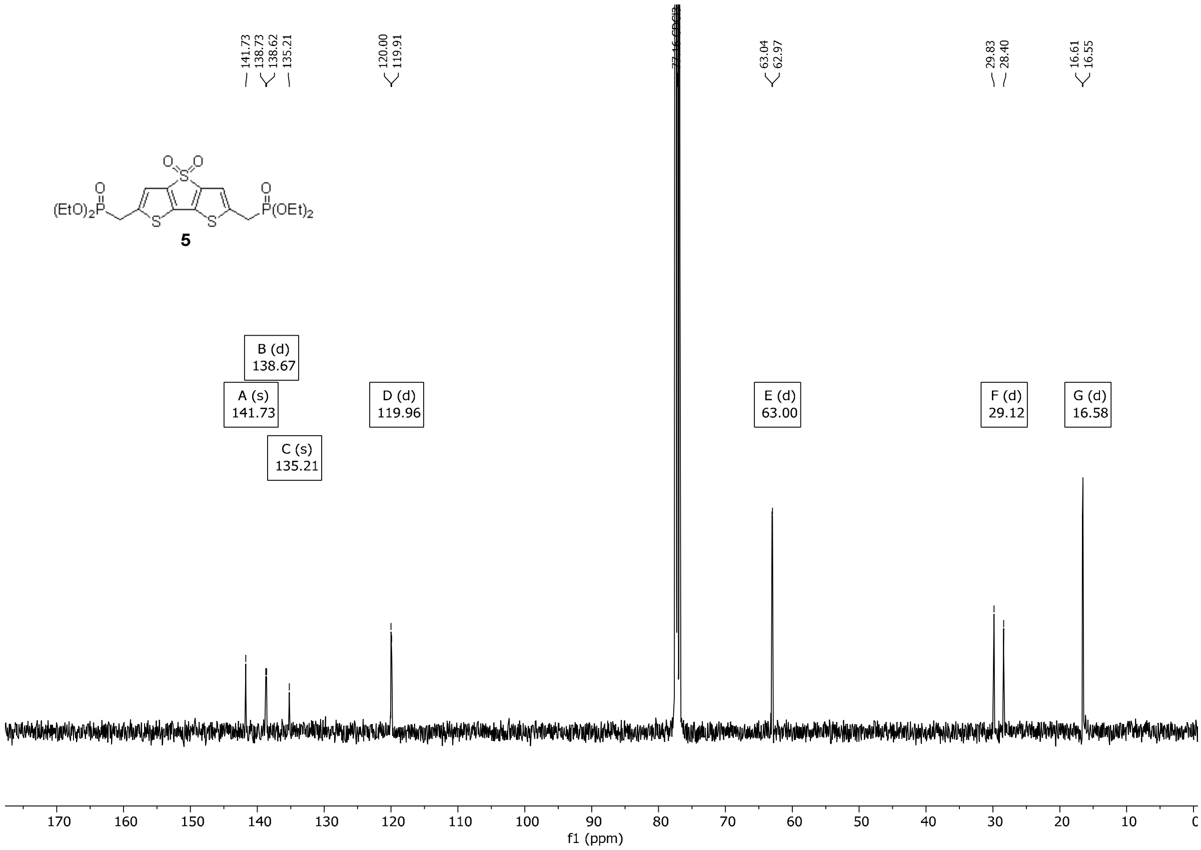
141.73
138.73
138.62
135.21
120.00
119.91
77.16 CDCl3
63.04
62.97
29.83
28.40
16.61
16.55
(EtO)2P
P(OEt)2
5
B (d)
138.67
A (s)
141.73
C (s)
135.21
D (d)
119.96
E (d)
63.00
F (d)
29.12
G (d)
16.58
f1 (ppm)

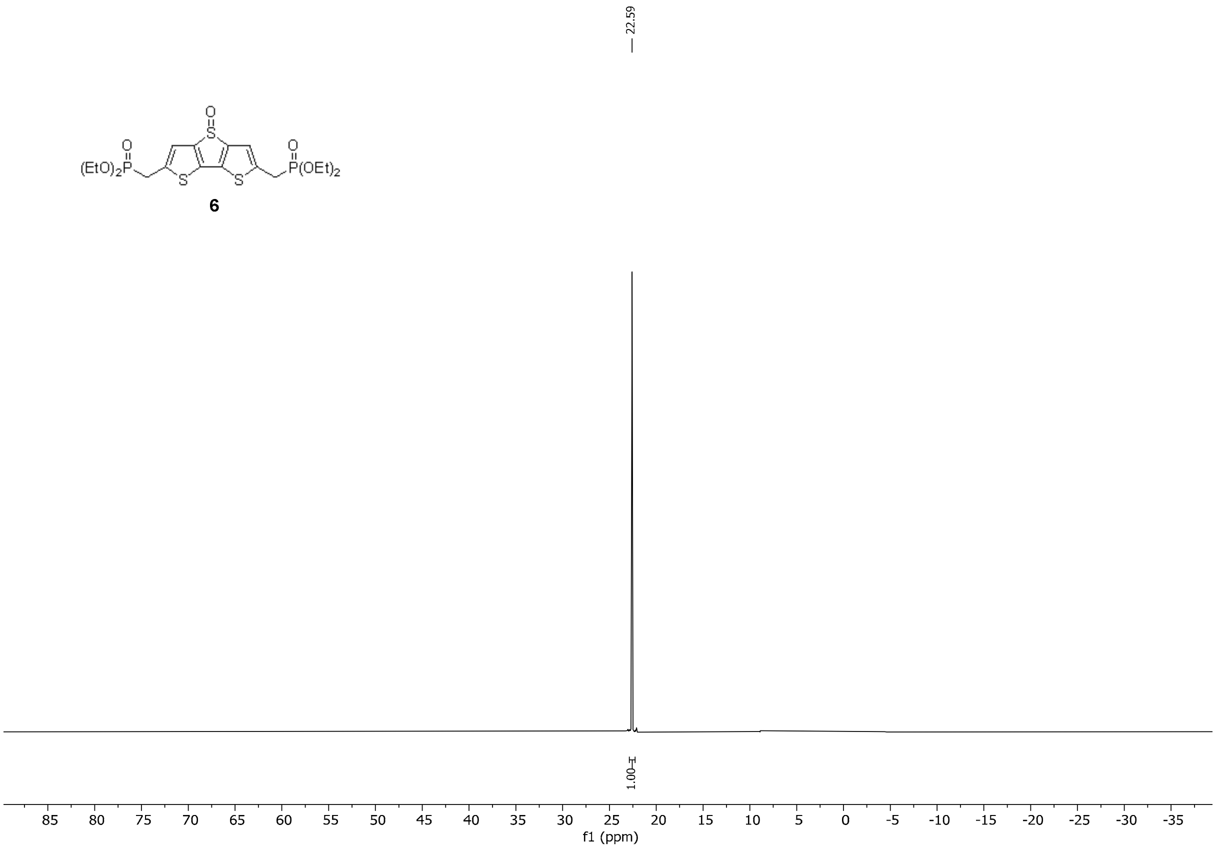
22.59
(EtO)2P(O)
P(OEt)2
6
1.00
85 80 75 70 65 60 55 50 45 40 35 30 25 20 15 10 5 0 -5 -10 -15 -20 -25 -30 -35
f1 (ppm)

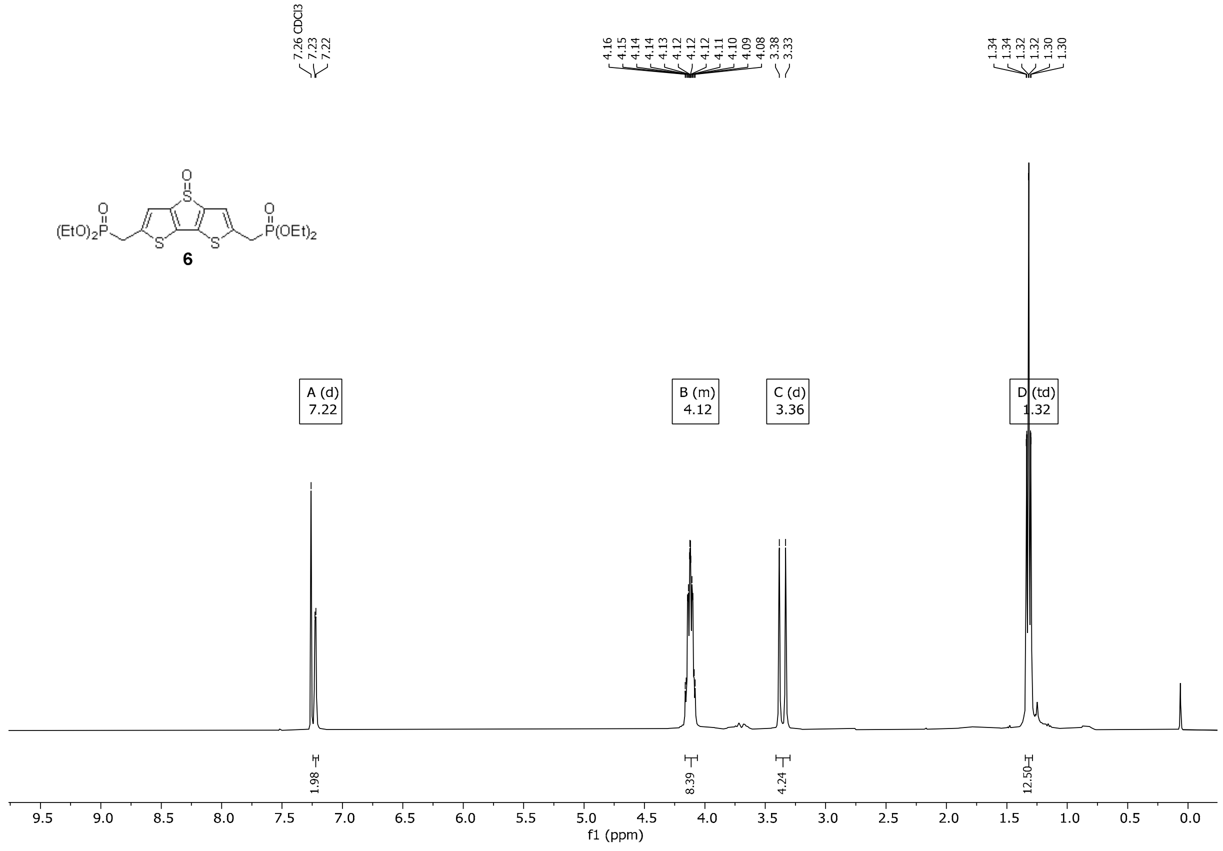
7.26 CDCl3
7.23
7.22
4.16 4.15 4.14 4.14 4.13 4.12 4.12 4.12 4.11 4.10 4.09 4.08
3.38 3.33
1.34 1.34 1.32 1.32 1.30 1.30
(EtO)2P(O)
P(OEt)2
6
A (d) 7.22
B (m) 4.12
C (d) 3.36
D (td) 1.32
1.98
8.39
4.24
12.50
9.5 9.0 8.5 8.0 7.5 7.0 6.5 6.0 5.5 5.0 4.5 4.0 3.5 3.0 2.5 2.0 1.5 1.0 0.5 0.0
f1 (ppm)

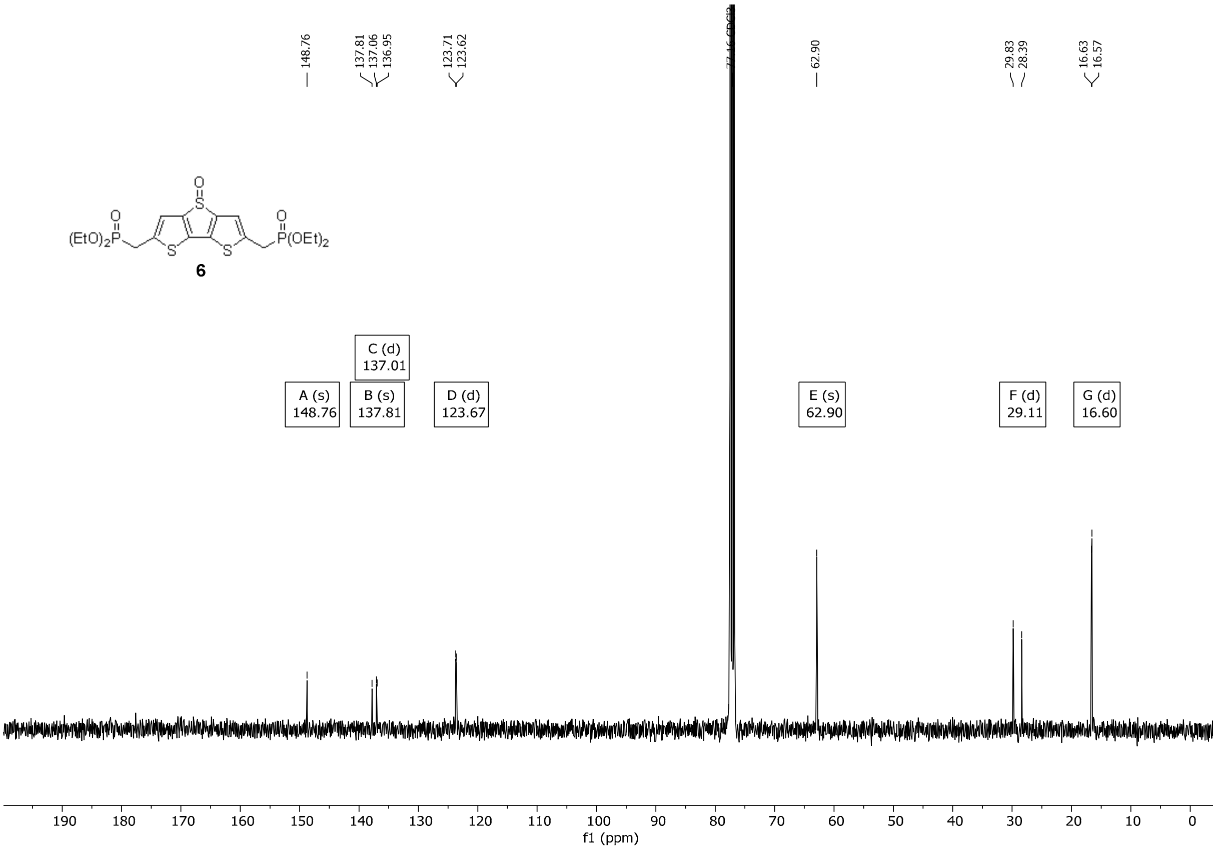
148.76
137.81
137.06
136.95
123.71
123.62
77.16 CDCl3
62.90
29.83
28.39
16.63
16.57
(EtO)2P
P(OEt)2
6
A (s)
148.76
B (s)
137.81
C (d)
137.01
D (d)
123.67
E (s)
62.90
F (d)
29.11
G (d)
16.60
190 180 170 160 150 140 130 120 110 100 90 80 70 60 50 40 30 20 10 0
f1 (ppm)

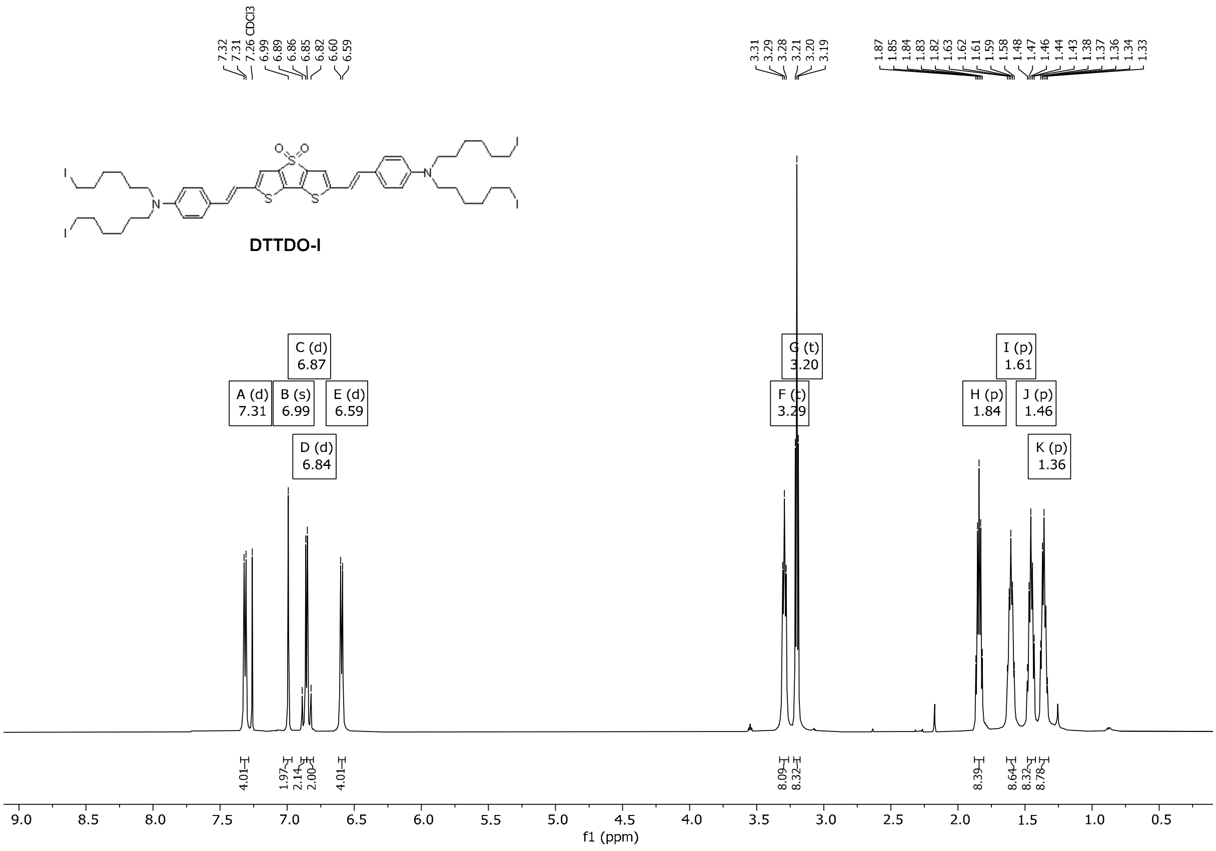
7.32
7.31
7.26 CDCl3
6.99
6.89
6.86
6.85
6.82
6.60
6.59
3.31
3.29
3.28
3.21
3.20
3.19
1.87
1.85
1.84
1.83
1.82
1.63
1.62
1.61
1.59
1.58
1.48
1.47
1.46
1.44
1.43
1.38
1.37
1.36
1.34
1.33
DTTDO-I
A (d) 7.31
B (s) 6.99
C (d) 6.87
D (d) 6.84
E (d) 6.59
F (t) 3.29
G (t) 3.20
H (p) 1.84
I (p) 1.61
J (p) 1.46
K (p) 1.36
4.01
1.97
2.14
2.00
4.01
8.09
8.32
8.39
8.64
8.32
8.78
f1 (ppm)

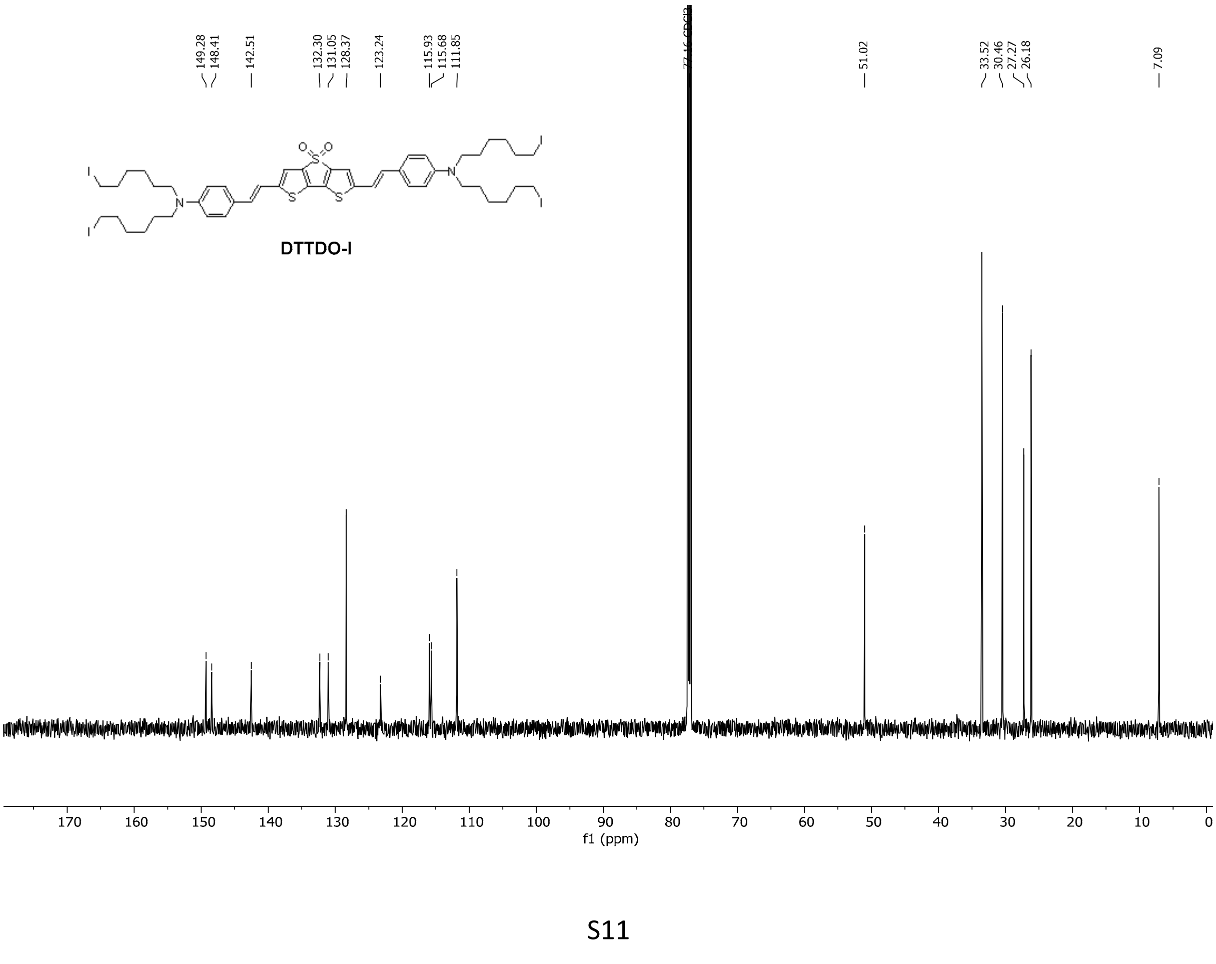
149.28
148.41
142.51
132.30
131.05
128.37
123.24
115.93
115.68
111.85
51.02
33.52
30.46
27.27
26.18
7.09
DTTDO-I
f1 (ppm)

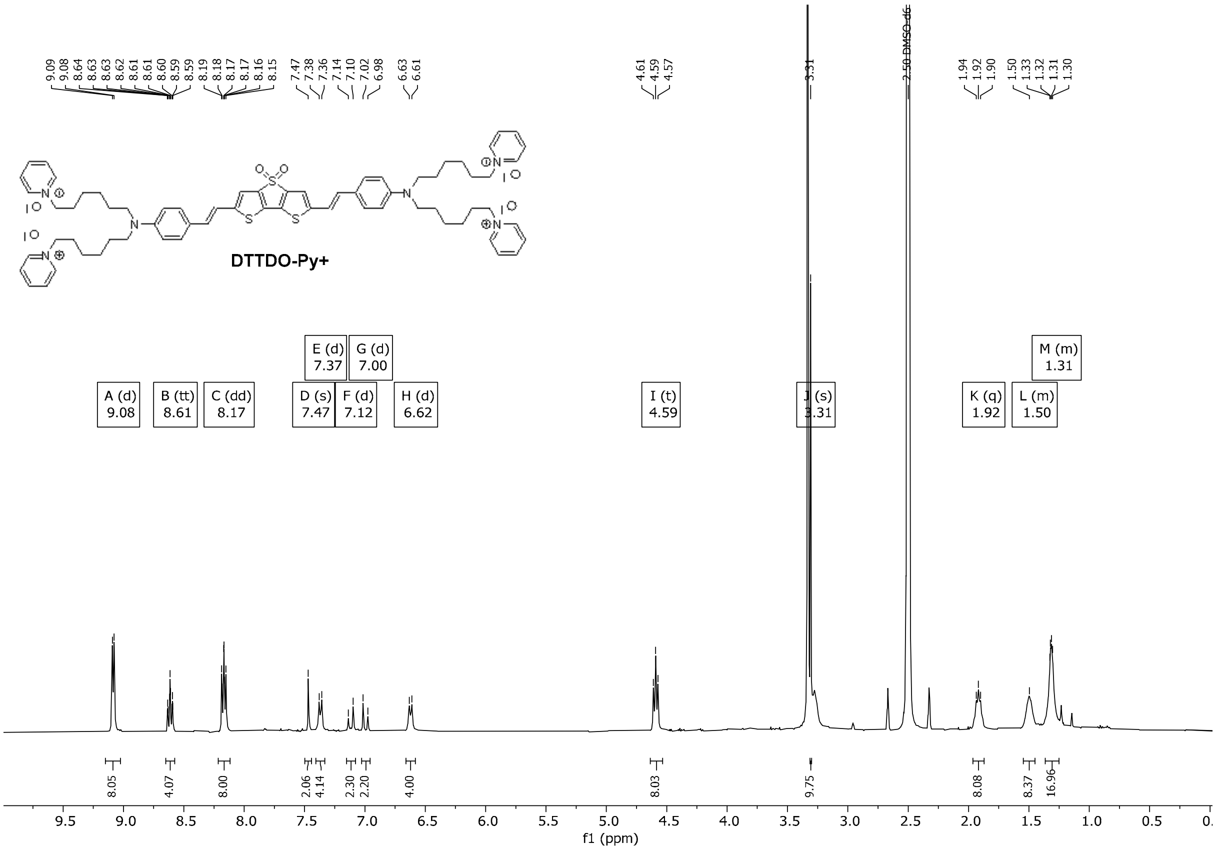
9.09
9.08
8.64
8.63
8.63
8.62
8.61
8.61
8.60
8.59
8.59
8.19
8.18
8.17
8.17
8.16
8.15
7.47
7.38
7.36
7.14
7.10
7.02
6.98
6.63
6.61
4.61
4.59
4.57
3.31
2.50 DMSO-d6
1.94
1.92
1.90
1.50
1.33
1.32
1.31
1.30
DTTDO-Py+
A (d) 9.08
B (tt) 8.61
C (dd) 8.17
D (s) 7.47
E (d) 7.37
F (d) 7.12
G (d) 7.00
H (d) 6.62
I (t) 4.59
J (s) 3.31
K (q) 1.92
L (m) 1.50
M (m) 1.31
8.05
4.07
8.00
2.06
4.14
2.30
2.20
4.00
8.03
9.75
8.08
8.37
16.96
f1 (ppm)

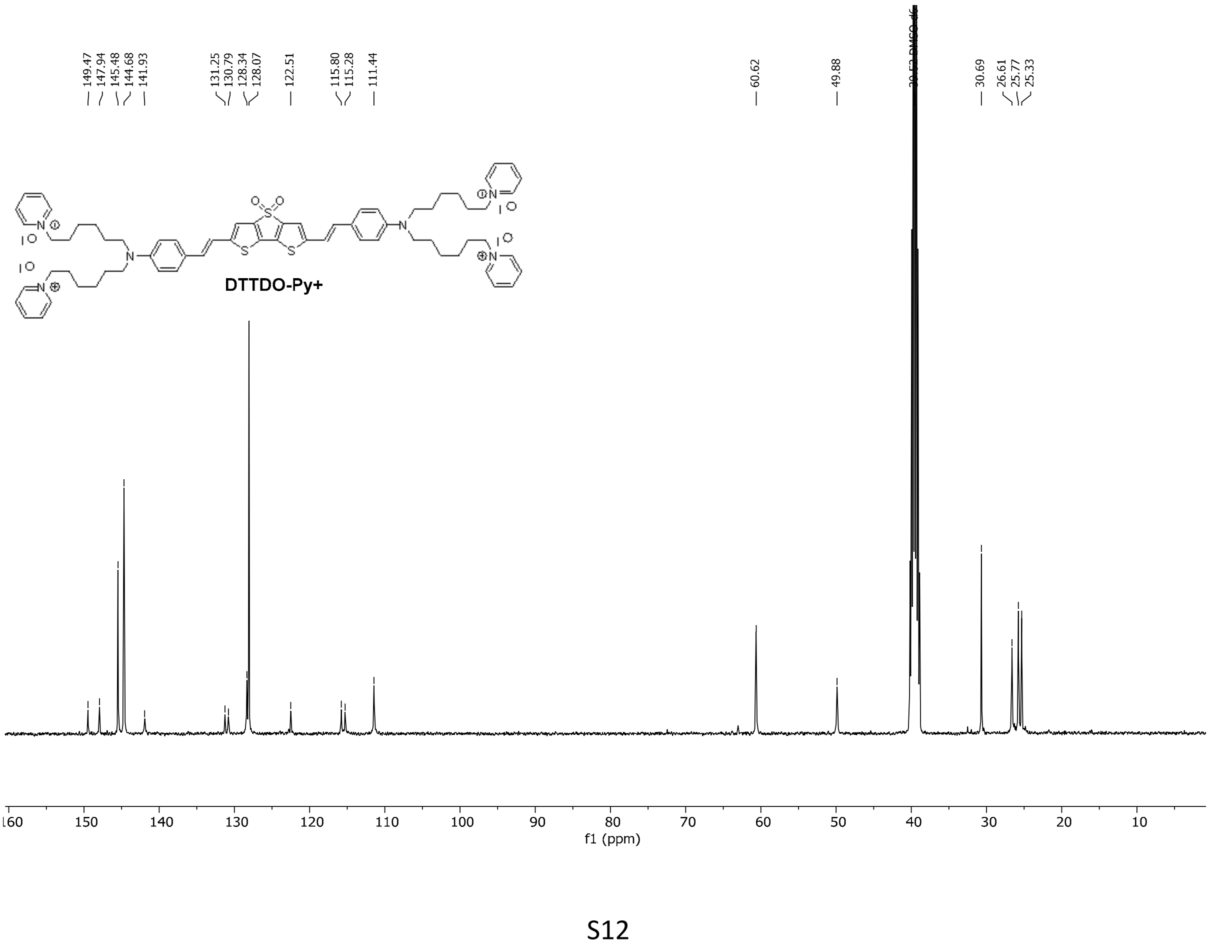
149.47
147.94
145.48
144.68
141.93
131.25
130.79
128.34
128.07
122.51
115.80
115.28
111.44
60.62
49.88
30.69
26.61
25.77
25.33
DTTDO-Py+
f1 (ppm)

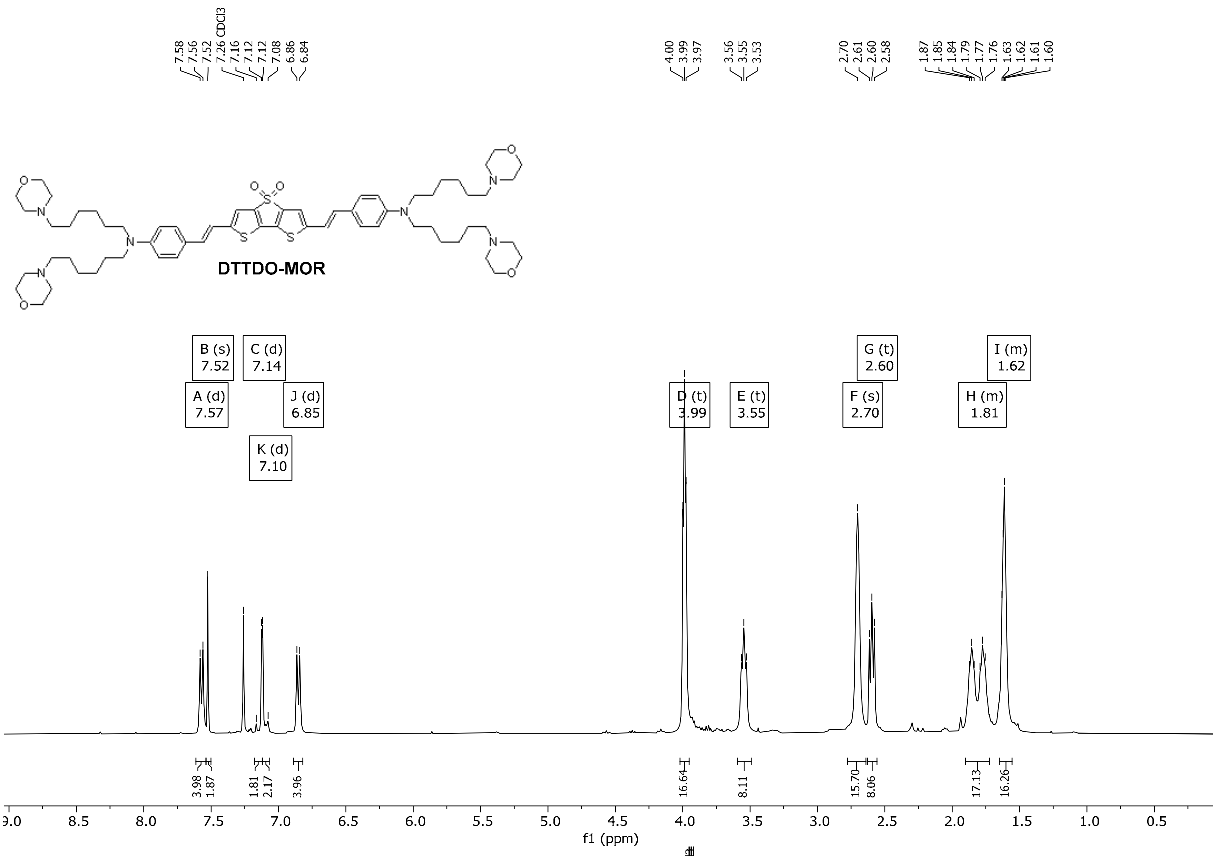
DTTDO-MOR
7.58
7.56
7.52
7.26 CDCl3
7.16
7.12
7.12
7.08
6.86
6.84
4.00
3.99
3.97
3.56
3.55
3.53
2.70
2.61
2.60
2.58
1.87
1.85
1.84
1.79
1.77
1.76
1.63
1.62
1.61
1.60
B (s) 7.52
C (d) 7.14
A (d) 7.57
J (d) 6.85
K (d) 7.10
G (t) 2.60
I (m) 1.62
D (t) 3.99
E (t) 3.55
F (s) 2.70
H (m) 1.81
3.98
1.87
1.81
2.17
3.96
16.64
8.11
15.70
8.06
17.13
16.26
f1 (ppm)

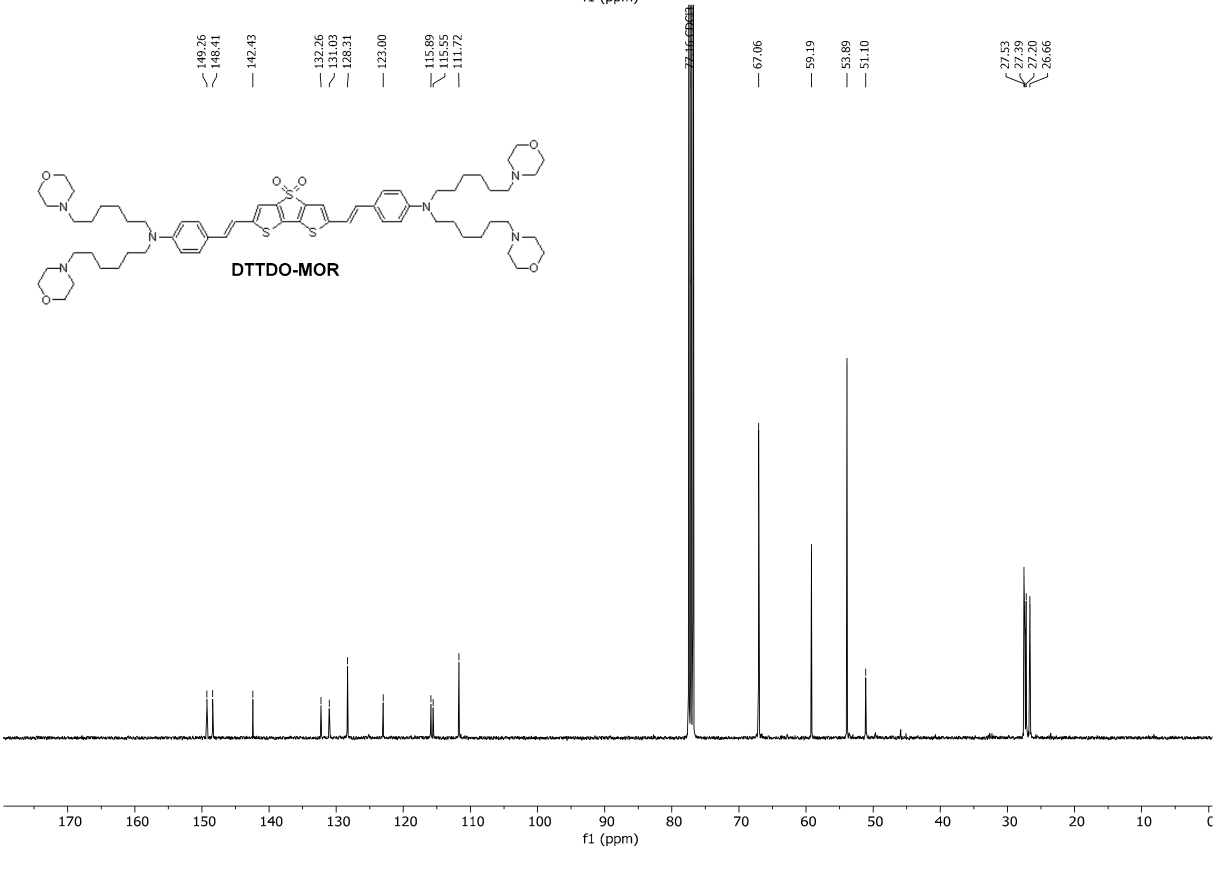
DTTDO-MOR
149.26
148.41
142.43
132.26
131.03
128.31
123.00
115.89
115.55
111.72
77.16 CDCl3
67.06
59.19
53.89
51.10
27.53
27.39
27.20
26.66
f1 (ppm)

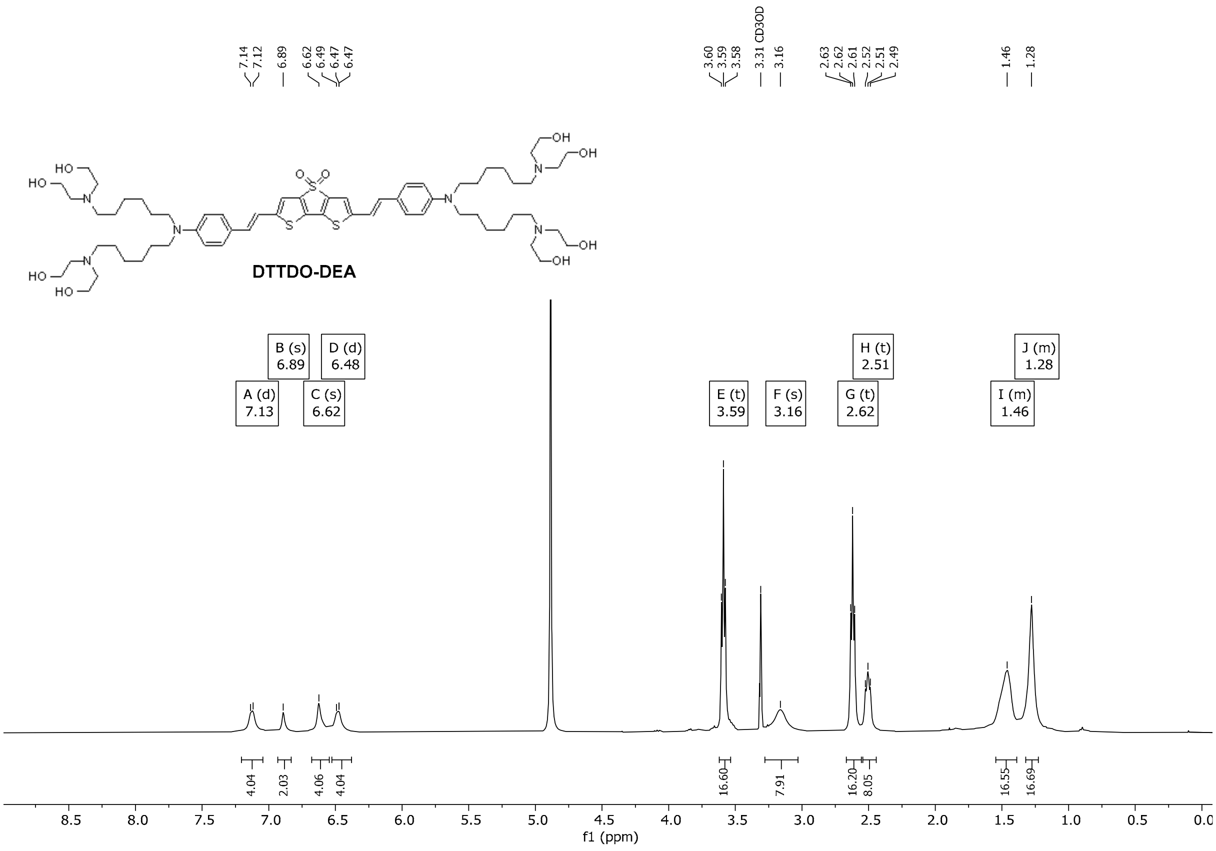
7.14
7.12
6.89
6.62
6.49
6.47
6.47
3.60
3.59
3.58
3.31 CD3OD
3.16
2.63
2.62
2.61
2.52
2.51
2.49
1.46
1.28
DTTDO-DEA
A (d) 7.13
B (s) 6.89
C (s) 6.62
D (d) 6.48
E (t) 3.59
F (s) 3.16
G (t) 2.62
H (t) 2.51
I (m) 1.46
J (m) 1.28
4.04
2.03
4.06
4.04
16.60
7.91
16.20
8.05
16.55
16.69
f1 (ppm)

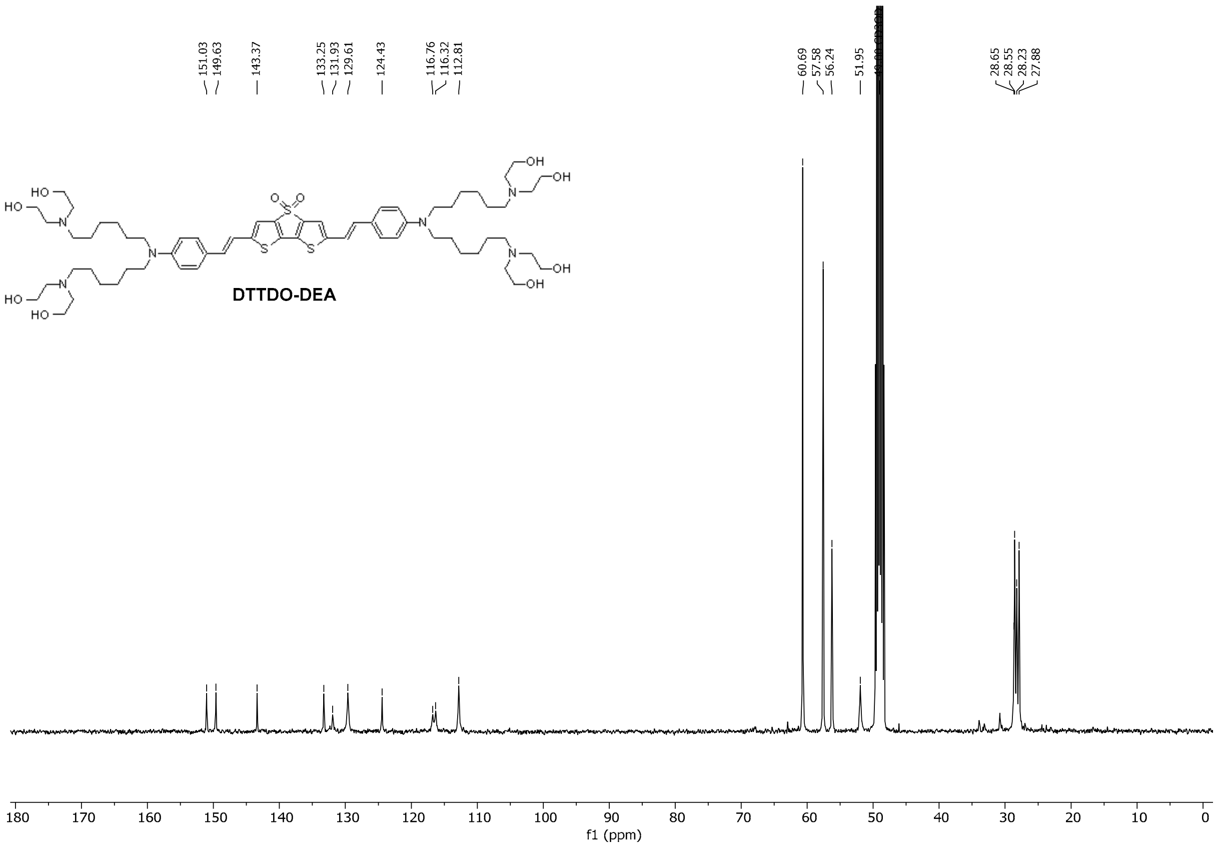
151.03
149.63
143.37
133.25
131.93
129.61
124.43
116.76
116.32
112.81
60.69
57.58
56.24
51.95
28.65
28.55
28.23
27.88
DTTDO-DEA
f1 (ppm)
